# Hypoxia-induced miR-653 enhances colorectal cancer progression by targeting circSETD3/KLF6 axis

**DOI:** 10.7150/jca.78865

**Published:** 2023-01-01

**Authors:** Qian Chen, Qingchun Deng, Yinglian Pan, Xiangwu Ding, Jing Liu

**Affiliations:** 1 Department of Gastroenterology, Wuhan Fourth Hospital, Wuhan 430033, China.; 2 Department of Gynecology, The Second Affiliated Hospital of Hainan Medical University, Haikou 570216, China.; 3 Department of Medical Oncology, The First Affiliated Hospital of Hainan Medical University, Haikou, 570102, China.; 4 Department of Neurology, Wuhan Fourth Hospital, Wuhan 430033, China.

**Keywords:** MiRNA-653, KLF6, Colorectal cancer, CircSETD3, TCGA

## Abstract

The present work focused on exploring the role and underlying molecular mechanism of action of the non-coding RNA (miRNA/circRNA) in colorectal cancer (CRC). Here, we found that miR-653 was dramatically upregulated in CRC tissues and cells. CRC Patients with high miR-653 level possessed poor prognosis. miR-653 elevated proliferation, migration, and invasion, meanwhile suppressed apoptosis of CRC cells. Furthermore, circSETD3 directly sponged miR-653 and negatively regulate miR-653 to affect proliferation, migration, invasion, and apoptosis of CRC cells. Moreover, miR-653 served as carcinoma-promoting gene via targeting KLF6, and circSETD3 knockdown significantly reversed the inhibitory effect of KLF6 overexpression on CRC cells. In addition, hypoxia obviously increased expression of miR-653. Knockdown of miR-653 decreased the effects of hypoxia on CRC cell proliferation, migration and invasion. Taken together, these findings indicated that circSETD3/miR-653/KLF6 axis may be an effective therapeutic target for CRC patients.

## Introduction

Colorectal cancer (CRC) is one of the most prevalent causes of cancer-related morbidity and mortality worldwide 1. Currently, surgical resection is still the most common curative treatment for patients with CRC 2. Despite great improvement in the treatment for CRC patients, including chemotherapy, radiotherapy, and immunotherapy, the postoperative result for CRC patients remains unfavorable due to the high incidence of cancer recurrence and metastasis 3. Therefore, it is urgently required to unearth the molecular pathways of CRC development and uncover effective hallmarks for predicting CRC patients' prognosis.

MiRNAs are a family of endogenous, small, and noncoding RNAs of nearly 22 nucleotides in length that play critical roles in gene regulation at post-transcriptional level for the initiation and development of human carcinomas 4-7. Increasing evidence suggested that dysregulated expression of miRNAs was associated with cancer progression 8, 9. Several reports have suggested that miRNA-653 was closely involved in cancer progression. For example, ciRs-6 upregulates March1 to suppress bladder cancer growth by sponging miR-653 10. PLK1S1 promoted tumor progression and sorafenib resistance in RCC through regulation of the miR‑653/CXCR5 axis 11. Meanwhile, in our study, miR-653 showed upregulation in CRC via high-throughput data and was correlated with dismal prognosis.

Various evidence indicated that hypoxia was a main feature of CRC, which may promote CRC progression 12, 13. A recent study reported that hypoxia-induced miRNA-590-5p promoted CRC progression via modulating matrix metalloproteinase activity 14, while associations between hypoxia and miR-653 in CRC remain unclear.

We investigated the oncogenic function of miR-653 in CRC and determined KLF6 as a direct target gene of miR-653. We found that the expression of miR-653 was negatively correlated with KLF6. Moreover, circSETD3 served as a sponge for miR-653, which further regulated KLF6 expression. Thus, the findings of the study revealed the oncogenic role of miR-653 in CRC tumorigenesis via targeting KLF6. Finally, the effects of hypoxia on miR-653 expression were explored. Our findings may provide novel insights into the treatment of CRC.

## Methods and Materials

### Bioinformatics data mining

Firstly, the raw CRC related miRNAs/mRNAs/circRNAs as well as eligible clinical information was achieved from Gene Expression Omnibus (GEO)/TCGA database. The inclusion criteria: 1) Designed experiments to screen for differential miRNAs/mRNAs/circRNAs between tumor and adjacent/non-tumor in humans. 2) Each dataset had at least 5 CRC samples and 5 control samples. Exclusion criteria: 1) Non-human studies. 2) Duplicated studies/datasets. 3) Non high-throughput data. Secondly, the CRC miRNAs and related clinical data were obtained from TCGA databases (TCGA-COAD and TCGA-READ datasets), which were then used to uncover the overlapped miRNAs with overall survival (OS)-related miRNAs. The miRNA raw expression count matrix was converted into transcripts per million (TPM) and normalized by Linear Models for Microarray (LIMMA) algorithm 15. Subsequently, the differential expression miRNAs (DEMs) between the CRC specimens and the non-tumor specimens were unearthed through Limma package 16 with an inclusion criteria of P<0.05 and logFC>1.3. In addition, the overlapped miRNAs were visualized by using Venn diagram based on vennDiagram package. Besides, the volcano map was adopted across ggplot2 package to weigh the expression level of the uncovered DEMs between the CRC cases and the non-tumor cases.

### Identification of miR-653 target genes

CircBank database was used to predict circRNAs interacting with miRNAs 17. MiRWalk2.0 18 was employed to explore the latent target genes of miR-653. The predicted target genes were attained if they were predicted simultaneously by 4 algorithms on the net comprising of miRWalk2.0 18, TargetScan6.2 19, miRanda 20 as well as RNA22 21. Next, the predicted targets were used to unearth overlapped genes with the DEGs in GSE50117 and GSE156355 datasets from GEO database. The DEGs between the CRC specimens and the non-tumor specimens were identified based on the above screening method 15. Finally, the identified targeted genes were adopted for the confirmation based on PCR and western blot.

### Cell Culture and Transfection

Colorectal cancer cell lines (SW480, LoVo) and normal colonic epithelial cell line (NCM460) were purchased from ATCC (Manassas, VA, USA). All cells were cultured in DMEM (HyClone, USA) containing 10% FBS. supplemented with 1% penicillin/streptomycin (Sigma-Aldrich, USA) in an atmosphere of 37 °C with 5% CO_2_. The hypoxic cell model was produced via culturing cells with 1% O_2_ and 5% CO_2_.

The miR-653 mimic, the mimics negative control (miR-NC) and miR-653 inhibitor were obtained from GenePharma (Shanghai, China). The pcDNA (Vector) and pcDNA-KLF6 overexpression (KLF6-OE) plasmids were achieved from GenePharma (Shanghai, China). Sequences for miR-653 and miR-653 inhibitor were cloned into lentiviral vectors; empty lentiviral vectors were applied as negative controls. Lipofectamine 2000 (Invitrogen, USA) was adopted to conduct transfection based on the manufacturer's protocol. The transfection efficacy was approximately 90% and was stable screened with puromycin. At 48h post‑transfection, cells were harvested for evaluation of transfection efficiency by qRT-PCR analysis.

### qRT-PCR

We employed TRIzol reagent (Invitrogen, Carlsbad, CA, USA) to extract the total RNA from CRC cells based on the manufacturer's protocol. Total RNA was employed to synthesize first-strand complementary DNA (cDNA) on the basis of the instructions of manufacturer (Invitrogen, Carlsbad, CA). Quantitative real-time PCR was implemented applying a RealMastcrMix kit (Tiangen Biotech) on the basis of the manufacturer's instructions. GAPDH was employed as the endogenous control for mRNA, and U6 functioned as the internal control for miRNA. The primers sequences applied for RT-qPCR were shown in **Table S1.** The 2^-ΔΔCt^ approach was employed to calculate fold changes.

### Western Blot

Total protein extraction of cell lines was conducted applying RIPA buffer (Pierce 89,900) according to the manufacturer's instructions. The proteins were separated on a 10% SDS-PAGE, which were then transferred onto PVDF membrane (GE Healthcare, NJ, USA). Next, the PVDF membrane was incubated with primary antibodies (GAPDH, 1:5000, ab8245, Abcam; KLF6, 1:1000, ab241385, Abcam; p21, 1:2000, ab109520, Abcam; E2F1, 1:4000, ab245308, Abcam; HIF1A, 1:1000, 20960-1-AP, Proteintech) at 4°C all through the night. Then, the membranes were washed in TBST buffer for 3 times and incubated with specific secondary antibody for 1 h. Finally, the membranes were visualized via an enhanced chemiluminescence (ECL) detection system (Millipore, USA).

### Luciferase reporter assay

To investigate the binding correlation between KLF6 3'UTR or circRNA and miR-653, 100 ng of pMIR-REPORT luciferase vector including KLF6 3'UTR or circRNA or a mutated type was used for co-transfection using miR-653 mimic or miR-653 inhibitor or miR-NC. Transfection was implemented based on the protocol of the manufacturer. Dual Luciferase Reporter Assay System (Promega) was employed to detect the luciferase activity in the cells according to manufacturer's instructions after 2 days transfection.

### Transwell assay

Transwell assay was performed to detect the invasion and migration capability of SW480 and LoVo cells. Transfected CRC cells were seeded in the upper chamber with serum-free DMEM, and the lower chamber was filled with DMEM containing 30% FBS. After incubating at 37°C for 24 h, cells that had migrated or invaded through the transwell membrane were fixed with 4% paraformaldehyde, then stained employing 0.1% crystal violet. A light microscope was utilized for the counting in transwell invasion and migration assay.

### Cell proliferation assay

For cell proliferation analysis, 5×10^3^ target cells were added in a 96-well plates and cultured at 37 °C for 1 to 5 days. At indicated time, the cells was cultured using 20 μl 3-(4,5)-dimethylthiahiazo (-z-y1)-3,5-diphenytetrazoliumromide (MTT, 5 mg/mL) for 4h at 37°C, which was dissolved applying 150μl DMSO (Sigma). The optical density (OD) at 450 nm was then assessed via a Microplate Reader (Bio-Rad, USA). For colony formation assay, 1.0 × 10^3^ treated cells were seeded in 6-well plates for approximately 14 days. These plates were washed with phosphate buffered saline (PBS) twice, fixed by 4% PFA and stained with 0.1% crystal violet solution for further analysis.

### Chromatin Immunoprecipitation (ChIP) Assay

ChIP analysis was conducted by using an EpiQuik ChIP Kit (Epigentek Group Inc.) based on the manufacturer's protocols. Cells were crosslinked using 2% formaldehyde for 10 min and then washed by using ice-cold PBS. After that, specimens were sheared via sonication on ice Lysates. Next, fragmented chromatin was immunoprecipitated applying antibodies against HIF-1α (HIF1A, 1:50, 20960-1-AP, Proteintech) or Flag together with Protein A/G PLUS-Agarose on a rotating platform overnight at 4°C. Eluted DNA was measured by qRT-PCR using the specific primers. Normal immunoglobulin G was exploited as a negative control. Anti-RNA Polymerase II was applied as positive control. Experiments were repeated at least 3 times.

### Statistical analysis

Each assay was conducted at least three times. All data was analyzed by GraphPad Prism 7.0. Data was displayed as mean ± standard deviation, in which the differences between two groups were assessed with Student's t-test while one-way ANOVA were conducted to test the difference among more than two groups. P value < 0.05 was considered significant.

## Results

### MiR-653 expression was upregulated in CRC and associated with poor prognosis

**Figure 1:**
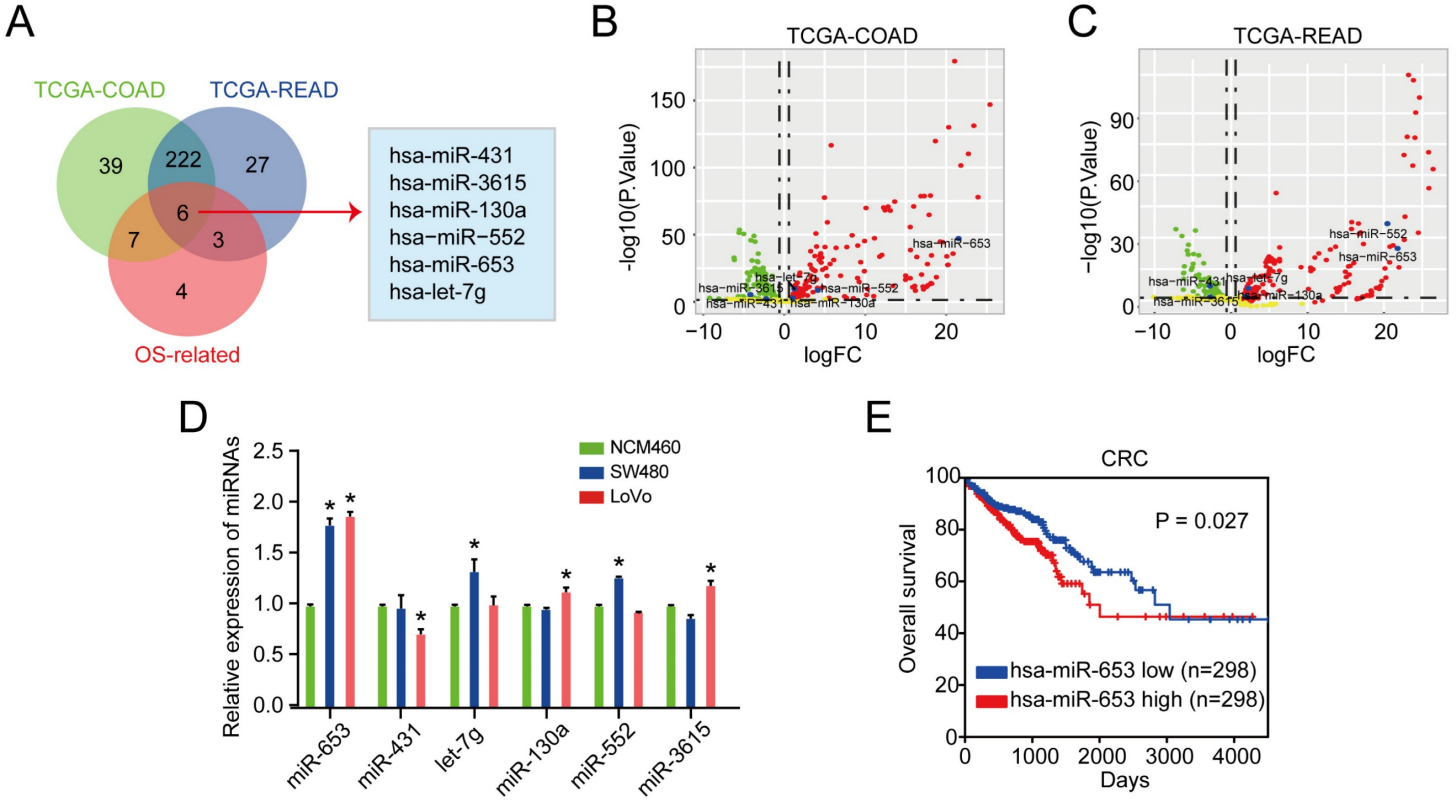
** MiR-653 expression was upregulated in CRC cell lines and tissues and associated with poor prognosis. (A)** 6 DEMs were determined via overlapping analysis of DEMs from the 3 sets. (**B & C**) Volcano plot of 6 DEMs mined from TCGA-COAD and TCGA-READ datasets. **(D)** qRT-PCR analysis of the 6 DEMs in SW480, LoVo and NCM460 cells. **(E)** Log-rank test for survival comparison. *P<0.05.

We identified 6 DEMs in CRC cases compared with normal cases according to the overlapped miRNAs analysis in TCGA database (TCGA-COAD, TCGA-READ datasets and OS-related miRNAs) (**Figure 1A**). The volcano plot of 6 DEMs in TCGA-COAD and TCGA-READ sets were also shown in **Figure 1B & 1C**, respectively. Then, we evaluated the uncovered 6 miRNAs expression in CRC cell lines. We observed that miR-653 was identified to be the most apparently upregulated molecule in both the DLD1 and SW48 cells than that in NCM460 cells (**Figure 1D**). Additionally, we explored whether the higher miR-653 expression was involved in CRC patients' survival via Kaplan-Meier model analysis. The analysis results revealed that patients with high expression levels of miR-653 had shorter OS (P < 0.05) (**Figure 1E**), suggestive of the good performance of miR-653 in OS prediction of CRC patients. The detailed clinic parameters of enrolled patients from public database was displayed in **Table S2-S6**.

### MiR-653 overexpression promotes proliferation, invasion and migration, cell cycle progression and inhibits the apoptosis of CRC cells

**Figure 2:**
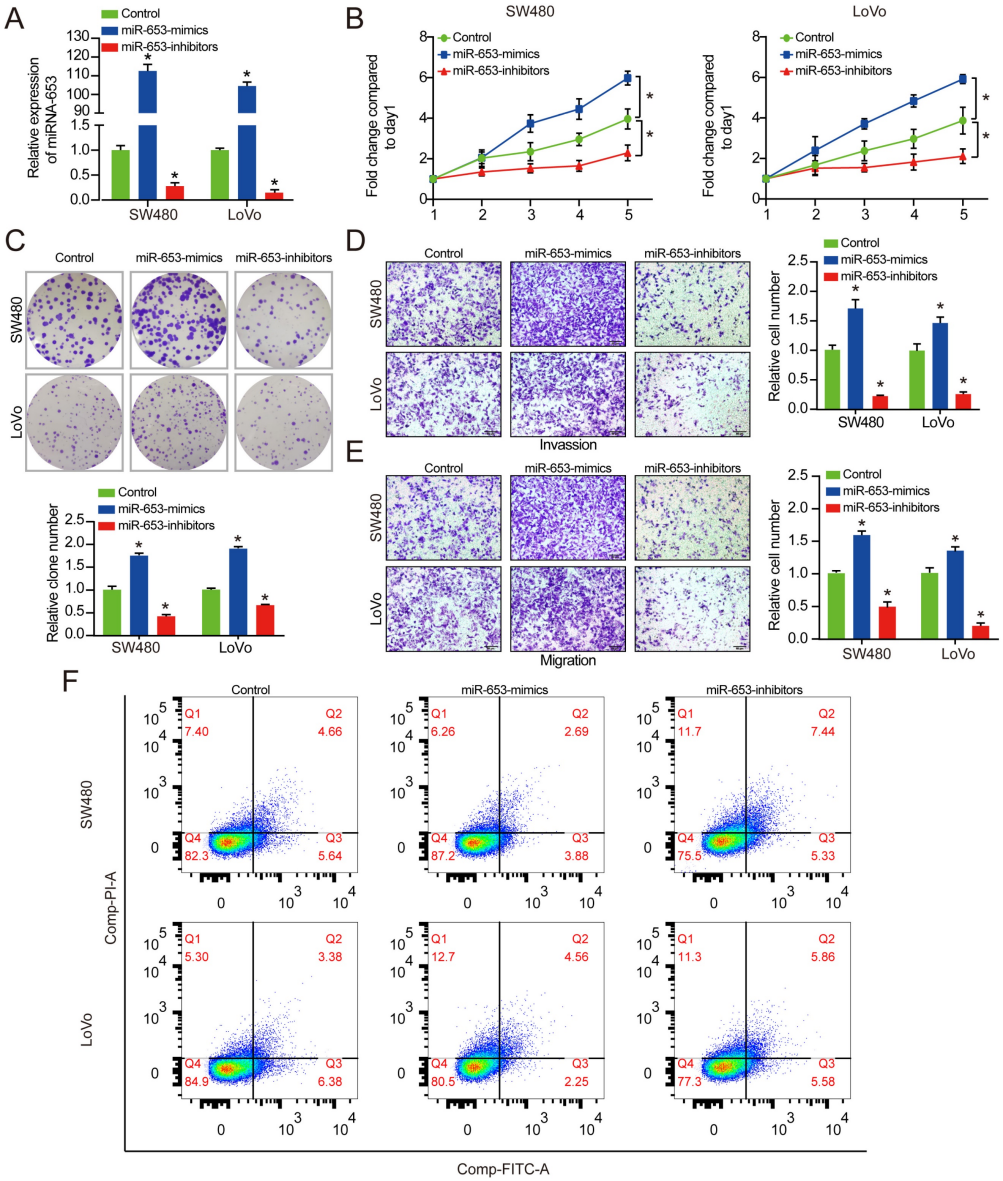
** MiR-653 overexpression promotes proliferation, invasion, migration, cell cycle and inhibits the apoptosis of CRC cells. (A)** qRT-PCR analysis of miR-653 expression with miR-653 mimic or miR-653 inhibitor in SW480 and LoVo cells. **(B & C)** Cell proliferative ability was measured by cell viability and colony formation with miR-653 overexpression or miR-653 knockdown in SW480 and LoVo cells.** (D**, **E)** Transwell assay was performed to assess cell invasion and migration ability with miR-653 overexpression or miR-653 knockdown in SW480 and LoVo cells. **(F)** The effect of miR-653 knockdown or overexpression on cell apoptosis in CRC cells. *P<0.05.

After that, we investigated the impact of miR-653 on the development and progression of CRC via overexpressing miR-653 or miR-653 knockdown. We found that expression of miR-653 was obviously increased by miR-653 mimics and expression of miR-653 was significantly suppressed by miR-653 knockdown (**Figure 2A**). As expected, miR-653 overexpression dramatically increased the proliferation, invasion as well as migration of CRC cells in SW480 and LoVo cells. On the other hand, miR-653 knockdown significantly decreased the proliferation, invasion as well as migration of CRC cells (**Figure 2B-E**), implying that upregulation of miR-653 expression in CRC cells could promote proliferation, invasion as well as migration of CRC cells. As indicated in **Figure 2F,** miR-653 overexpression significantly inhibited CRC cells apoptosis and miR-653 knockdown obviously promoted CRC cells apoptosis.

### The expression of hsa_circ_000567 was negatively correlated with the expression of miR-653

**Figure 3:**
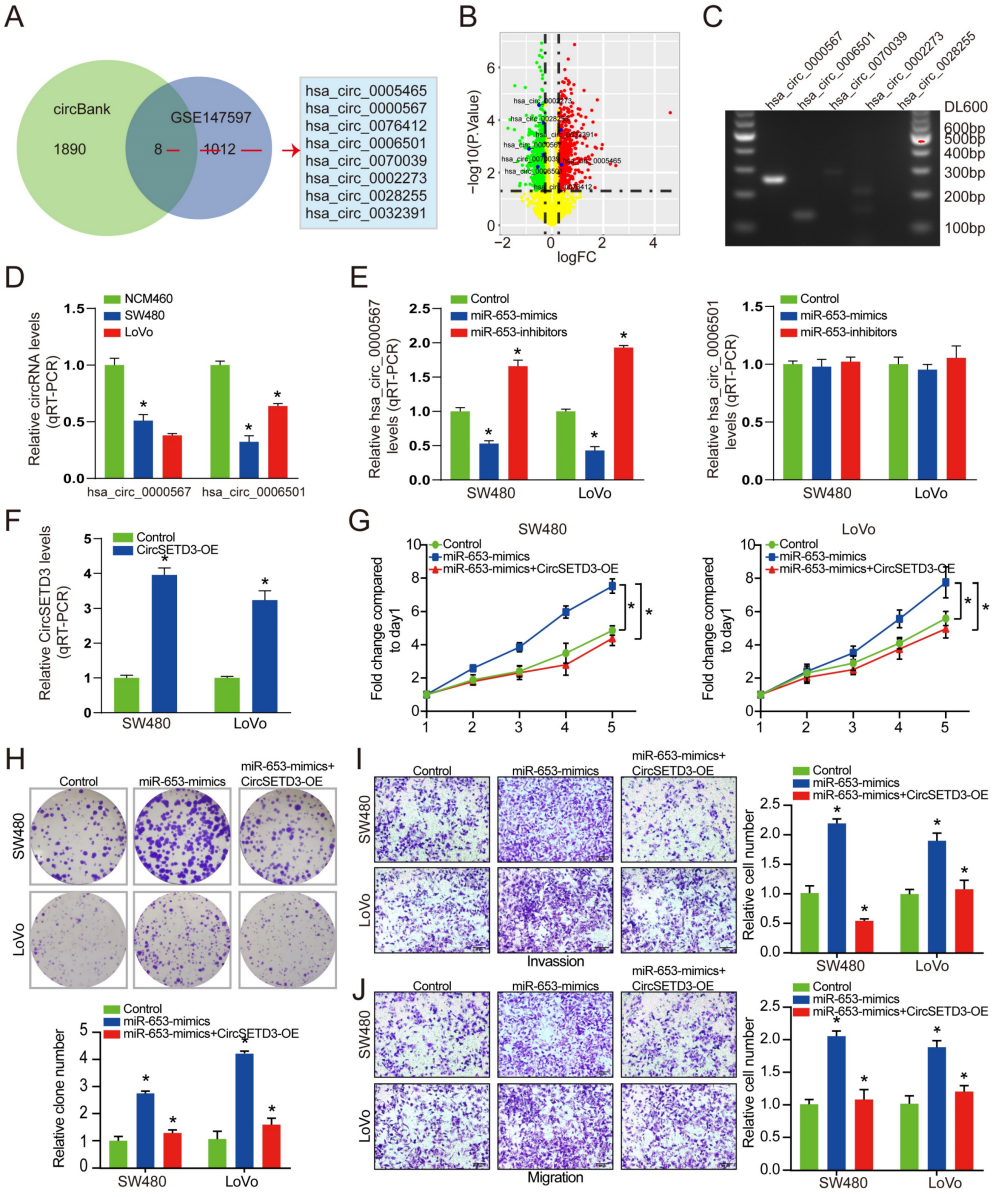
** The expression of hsa_circ_000567 was negatively correlated with the expression of miR-653. (A)** Eight DECs were determined via overlapping predicted circRNAs from circBank with DECs from GSE147597 dataset. **(B)** The volcano plot of the eight DECs in GSE147597 dataset.** (C)** RT-PCR assay with divergent primers showing the screened circRNAs in cultured SW480 cells. **(D)** qRT-PCR analysis of hsa_circ_0000567 and hsa_circ_000651 expression in SW480, LoVo and NCM460 cells. **(E)** qRT-PCR analysis of hsa_circ_0000567 and hsa_circ_000651 expression with miR-653 overexpression or miR-653 knockdown in SW480 and LoVo cells. **(F)** qRT-PCR analysis of circSETD3 expression with circSETD3 overexpression in SW480 and LoVo cells. **(G, H)** Cell proliferative ability was measured by cell viability and colony formation with miR-653 overexpression or miR-653 overexpression + circSETD3 overexpression in SW480 and LoVo cells. **(I, J)** Transwell assay was performed to assess cell invasion and migration ability with miR-653 overexpression or miR-653 overexpression + circSETD3 overexpression in SW480 and LoVo cells. *P<0.05.

Upon uncovering that miR-653 promote the proliferation, invasion and migration of CRC cells, we next investigated the possible mechanisms underlying these functions. Emerging evidence has shown that some circRNAs regulate the expression patterns and functions of miRNAs. Hence, it is reasonable to speculate that some circRNAs could regulate the progression of CRC cells by interacting with miR-653. Therefore, we analyzed the circRNA expression profiles in CRC from publicly available data, and identified eight differentially expressed circRNAs, three of which were upregulated and five were downregulated (**Figure 3A & 3B**). Interestingly, our PCR results showed that only hsa_circ_0000567 and hsa_circ_0006501 could be amplified by cDNA templates in CRC cells (**Figure 3C**). We performed Sanger sequencing using the PCR products of these two circRNAs, and the results identified these two circRNAs that were consistent with the sequence information in the circBase database (**Figure S1A & S1B**). Next, the expression of hsa_circ_0000567 and hsa_circ_0006501 were detected by qRT-PCR in SW480, LoVo and NCM460 cell lines, respectively. Results confirmed that hsa_circ_0000567 and hsa_circ_0006501 were significantly downregulated in CRC cells. Then, the relative expression of these two circRNAs were determined in miR-653 overexpression and knockdown system. As shown in **Figure 3E**, after transfected with miR-653 mimics or inhibitors, the level of hsa_circ_0000567 was negatively affected, while there was no change in expression of hsa_circ_0006501. Besides, the resistance to digestion via RNase R exonuclease indicated that hsa_circ_0000567 had a circular RNA structure (**Figure S1C**). Based on these data, we hypothesized that miR-653 could be a target of hsa_circ_0000567 (circSETD3). Additionally, the transfection efficiency of the circSETD3-overexpressing vector in CRC cells was verified by qRT-PCR (**Figure 3F**). Moreover, we found that transfection of SW480 and LoVo cells with miR-653 mimics promoted cell proliferation, invasion and migration. These effects were significantly reversed by circSETD3 overexpression in SW480 and LoVo cells (**Figure 3G-3J**). Taken together, these experiments indicate that circSETD3 may function as a sponge for miR-653 in CRC cells.

### KLF6 is a direct target of miR-653 in CRC cells and restoration of KLF6 reverse the promoting effects of miR-653

**Figure 4:**
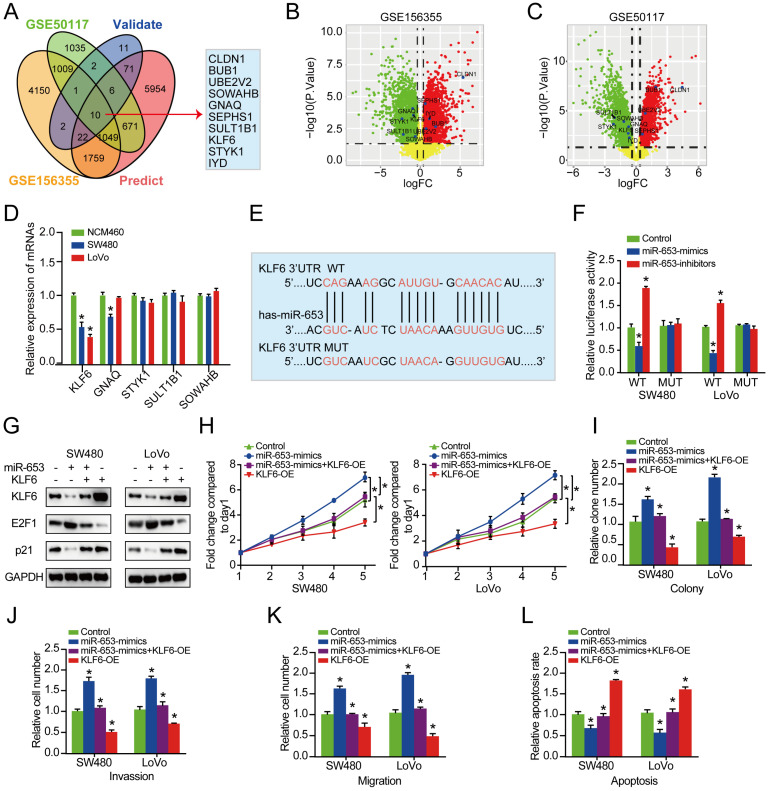
** KLF6 is a direct target of miR-653 and restoration of KLF6 reverses the promoting effects of miR-653 in CRC cells. (A)** Overlapping analysis of DEGs from GSE50117, GSE156355, predicted and validated target genes uncovered 10 consensus target genes.** (B, C)** Volcano plot of 10 DEGs unearthed from GSE50117 and GSE156355, respectively; logFC represents log10(fold change). **(D)** qRT-PCR analysis of the down-regulated target genes in NCM460, SW480 and LoVo cells. **(E)** miR-653 binding site within the 3'-UTR of KLF6 was analyzed. (**F**) pMIR-REPORT luciferase vector consisting of KLF6 3'UTR or a mutated form was co-transfected in SW480 and LoVo cells with miR-653 mimic or miR-653-inhibitor or miR-NC. Firefly luciferase activity was detected and normalized with Renilla luciferase activity; WT: wild type; MUT: mutation type. **(G)** Western blot analysis for KLF6, P21 and E2F1 in SW480 and LoVo cells with miR-653 overexpression or miR-653 overexpression + KLF6 overexpression or KLF6 overexpression. **(H, I)** Cell proliferative ability was measured by cell viability and colony formation with miR-653 overexpression or miR-653 overexpression + KLF6 overexpression or KLF6 overexpression in DLD1 and SW48 cells.** (J, K)** Cell invasion and migration ability were assessed by transwell assay with miR-653 overexpression or miR-653 overexpression + KLF6 overexpression or KLF6 overexpression in DLD1 and SW48 cells. **(L)** The effect of KLF6 and miR-653 on cell apoptosis in CRC cells. *P<0.05.

Next, we explored the potential downstream target mRNAs of circSETD3/miR-653 axis. 10 consensus target genes were unearthed through overlapping analysis of DEGs from GSE50117 and GSE156355 with predicted and validated target genes using miRWalk database (**Figure 4A**). Among the 10 identified target genes, 5 consensus target genes in CRC tissues were both downregulated in GSE156355 and GSE50117 (**Figure 4B & 4C**). In addition, among the 5 reduced genes, KLF6 expression was the most significantly decreased in both SW480 and LoVo cells than that in NCM460 cells (**Figure 4D**). Next, the binding sites of KLF6 mRNA with miR-653 were predicted via TargetScan (http://www.targetscan.org) online databases. We discovered that the 3'-UTR of KLF6 mRNA had the complementary sequence of miR-653 based on online available database (**Figure 4E**). Therefore, KLF6 was used for further experimental confirmation across a luciferase reporter assay. Importantly, the result manifested that miR-653 overexpression prominently reduced the fluorescence intensity in cells transfected with WT-KLF6 luciferase reporter plasmid, whereas, miR-653 knockdown clearly enhanced the fluorescence intensity in the WT-KLF6 construction but not MUT-KLF6 construction (**Figure 4F**). These results suggested the direct interaction between miR-653 and the 3'UTR of KLF6 mRNA. In addition, miR-653 overexpression significantly decreased KLF6 protein expression in SW480 and LoVo cells (**Figure 4G**). Meanwhile, Previous studies have identified P21 and E2F1 as the target genes of KLF6 22, 23. Western blot results showed that miR-653 overexpression obviously increased E2F1 expression and significantly reduced KLF6 and P21 the expression. While miR-653 knockdown yielded a reverse result (**Figure 4G**). Moreover, high miR-653 expression prominently promoted the proliferation, invasion and migration of CRC cells, which was obviously reversed by KLF6 overexpression (**Figure 4H-K**). The result also suggested that increased miR-653 expression significantly suppressed apoptosis of CRC cells, which was apparently rescued by KLF6 overexpression (**Figure 4L**). We speculated that miR-653 might exert its function in CRC cells by directly targeting KLF6.

### KLF6 is regulated by circSETD3 in CRC cells

**Figure 5:**
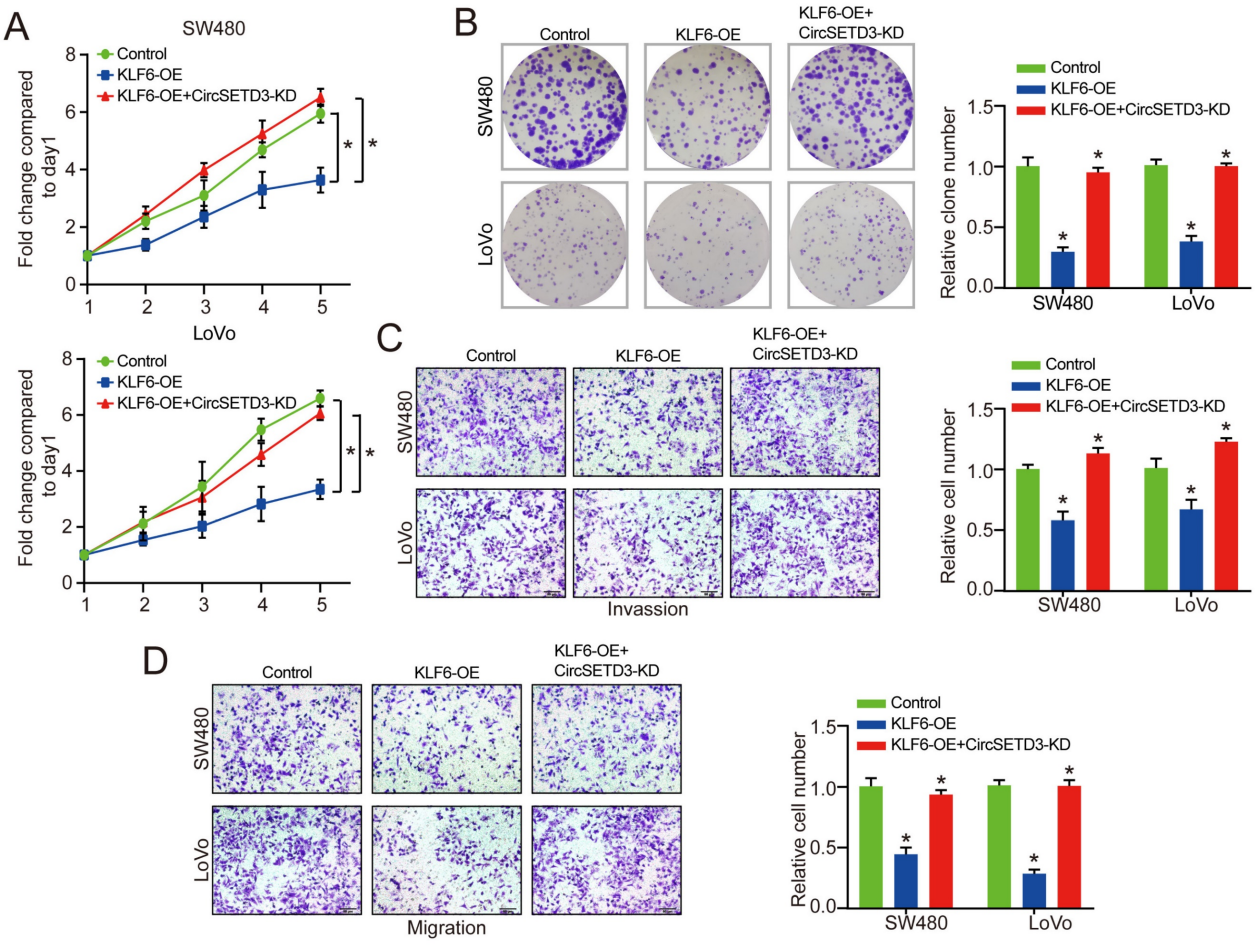
** KLF6 is regulated by circSETD3 in CRC cells.** (**A, B**) Cell proliferative ability was measured by cell viability and colony formation with KLF6 overexpression or KLF6 overexpression + circSETD3 knockdown in SW480 and LoVo cells. (**C, D**) Cell invasion and migration ability were assessed by transwell assay with miR-653 overexpression or miR-653 overexpression + KLF6 overexpression or KLF6 overexpression in SW480 and LoVo cells. *P<0.05.

Next, the KLF6-overexpression plasmid was co-transfected into CRC cells to verify the effect of LPP on proliferation, invasion, and migration of circSETD3 knockdown CRC cells. The MTT and colony formation results indicated that KLF6 overexpression decreased the proliferation of CRC cells, which was partially reversed by circSETD3 knockdown (**Figure 5A & 5B**). Similarly, in transwell invasion and migration assay, the results demonstrated that KLF6 overexpression could inhibit cell migration and invasion abilities, however, this inhibitory effect was attenuated by downregulation of circSETD3 in SW480 and LoVo cells (**Figure 5C & 5D**).

### MiR-653 expression is transcriptionally promoted by HIF-1A under hypoxia

**Figure 6:**
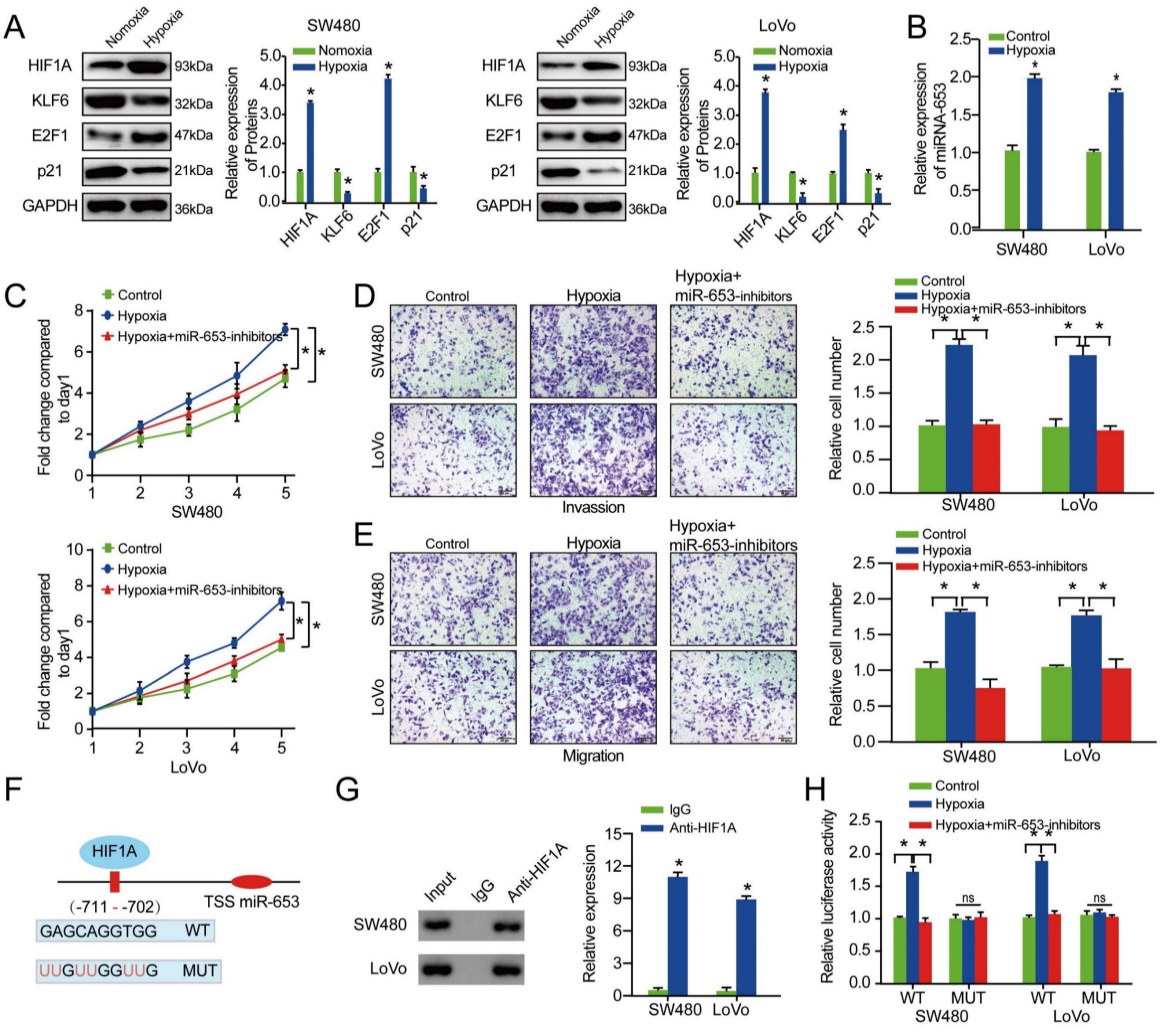
** MiR-653 expression is transcriptionally promoted by HIF-1A under hypoxia**. (**A**) The effect of hypoxia on the expression of HIF-1A, KLF6, P21 and E2F1 was assessed via western blot analysis for in SW480 and LoVo cells. (**B**) MiR-653 expression was detected in SW480 and LoVo cells under hypoxia. (**C**) Cell proliferative ability was measured by cell viability with miR-653 knockdown in SW480 and LoVo cells under hypoxia. (**D, E**) Hypoxia obviously elevated cell invasion and migratory ability of SW480 and LoVo cells, which was significantly rescued by miR-653 knockdown via transwell assay. (**F**) A schematic suggestive of the proximal region of the miR-653 promoter was bound by HIF-1A. (**G**) ChIP was conducted applying an anti-HIF-1A antibody to confirm the binding between HIF-1A and the miR-653 promoter in SW480 and LoVo cells. (**H**) Luciferase activity was obviously increased in the WT miR-653 promoter under hypoxia, which was significantly rescued by miR-653 knockdown. While, these impacts were not found when HIF1A binding motif at -711 ~ -702bp were mutated. *P<0.05, **P<0.01.

Various studies revealed HIF1A as a critical transcription factor induced by hypoxia, which could target to the hypoxia response elements (HRE) within target genes' promoter to regulate it transcriptionally 24, 25. To explore the effect of hypoxia on miR-653-KLF6 axis, western blot assay was performed to explore the relative expression of HIF1A, KLF6, P21 and E2F1 under hypoxia. The result indicated that HIF1A and E2F1 expression were obviously increased while KLF6 and P21 expression were significantly decreased under hypoxia in both of the CRC cells (**Figure 6A**). As indicated in **Figure 6B**, the result exhibited that miR-653 expression level was apparently upregulated by hypoxia. The results also suggested that hypoxia could promote proliferation, invasion as well as migration of CRC cells, which could be reversed by miR-653 knockdown (**Figure 6C-E**).

Then, ChIP assay was performed to explore whether HIF1A transcriptionally modulated miR-653 expression. We analyzed the underlying HRE in upstream of the miR-653 sequence according to the JASPAR bioinformatics website and found a HRE inside the presumptive miR-653 promoter region. ChIP assays verified that the region at -711 ~ -702bp upstream from transcription start site of the pre-miR-653 was occupied by HIF1A (**Figure 6F, G**). In addition, increased luciferase activity in the WT miR-653 promoter was discovered under hypoxia, which was significantly rescued by miR-653 Knockdown. These impacts were not found when HIF1A binding motif at -711 ~ -702bp were mutated (**Figure 6H**). These results suggested that HIF1A directly promoted miR-653 at transcript levels through directly targeting the promoter region of miR-653. These data concluded that hypoxia-induced miR-653 enhanced colorectal cancer growth and metastasis via targeting circSETD3/KLF6 axis.

## Discussion

Numerous studies demonstrated that miRNAs were closely correlated with tumorigenesis 26, 27. Notably, numerous miRNAs have been discovered to be correlated with treatment of plenty of cancers 28, 29. Increasing studies indicated that miR-653 was closely involved in numerous carcinomas 30, 31. While, fewer studies have investigated the effect of miR-653 on CRC development. We analyzed the miRNA microarray data from TCGA database and uncovered that miR-653 was remarkably elevated in CRC samples, suggesting that miR-653 may function as an oncogenic miRNA in CRC progression.

To explore the molecular mechanisms of miR-653 in CRC, we predicted candidate target genes of miR-653 according to public available database and KLF6 was determined as a direct target of miR-653 through luciferase reporter assay. Our findings showed that high miR-653 expression prominently promoted the proliferation, invasion and migration of CRC cells, which was clearly reversed by KLF6 overexpression. Kaplan-Meier analysis for CRC patients from TCGA database suggested that CRC patients with high miR-653 level yielded an apparently shorter OS compared to those with high miR-653 level. Moreover, circSETD3 was downregulate in both CRC tissue and cells. Overexpression of circSETD3 can block the promoting role of miR-653 in CRC. Knockdown of circSETD3 reversed the inhibitory effect of KLF6 overexpression. After that, we explored the effect of hypoxia on the progression of CRC cells and found that hypoxia could promote proliferation, invasion as well as migration of CRC cells, which could be reversed by miR-653 knockdown. Finally, our ChIP assay confirmed that HIF1A regulated miR-653 directly at transcription level.

In addition, KLF6 has been reported to be a tumor inhibitor in several cancer kinds. For example, Gao et al. suggested that KLF6 suppressed metastasis of clear cell renal cell carcinoma through transcriptional repression of E2F1 23. Qing et al. found that down-regulation of KLF6 might play a critical role in the carcinogenesis and progression of gastric cancer 32. The above studies were consistent with the result in our study. Furthermore, we concluded that miR-653 exerts its function, at least to a certain extent, via KLF6 down-regulation.

Increasing evidence suggests circRNAs exert critical functions in CRC progression via sponging miRNAs 33, 34. For instance, CircRNA_0000392 as sponges miR-193a-5p via its miR-193a-5p targeting sites and regulates CRC progression 34. CircHIPK3 is significantly upregulated in CRC tissue and CircHIPK3 knockdown suppresses the proliferation and increased apoptosis of CRC cells by sponging miR-7 35. According to our data, we confirm that circSETD3 regulates KLF6 via sponging miR-653, Therefore, our study indicates that circSETD3 can sponge miR-653 and subsequently regulate miR-653 targeting genes.

In conclusion, our study unveiled the critical role of miR-653 in the CRC progress, especially in hypoxia. Mechanistically, circSETD3 harbor miR-653, acting as miRNA sponge, and then miR-653 targeted the KLF6. Moreover, HIF1α could bind with the promoter of miR-653 and promoted its transcription. The finding illustrates the vital role of circSETD3/miR-653/KLF6 axis in CRC tumorigenesis.

## Supplementary Material

Supplementary figure and tables.
