# Supplementary material for: Hypoxia-induced miR-653 enhances colorectal cancer progression by targeting circSETD3/KLF6 axis
Source: J Cancer. 2023 Jan 1;14(1):163–73. doi: 10.7150/jca.78865 (PMC9809325; doi:10.7150/jca.78865)
Supplement: Supplementary file 1 — Supplementary figure and tables. [file jcav14p0163s1.pdf]

Figure S1. Characteristics of the CircSETD3.

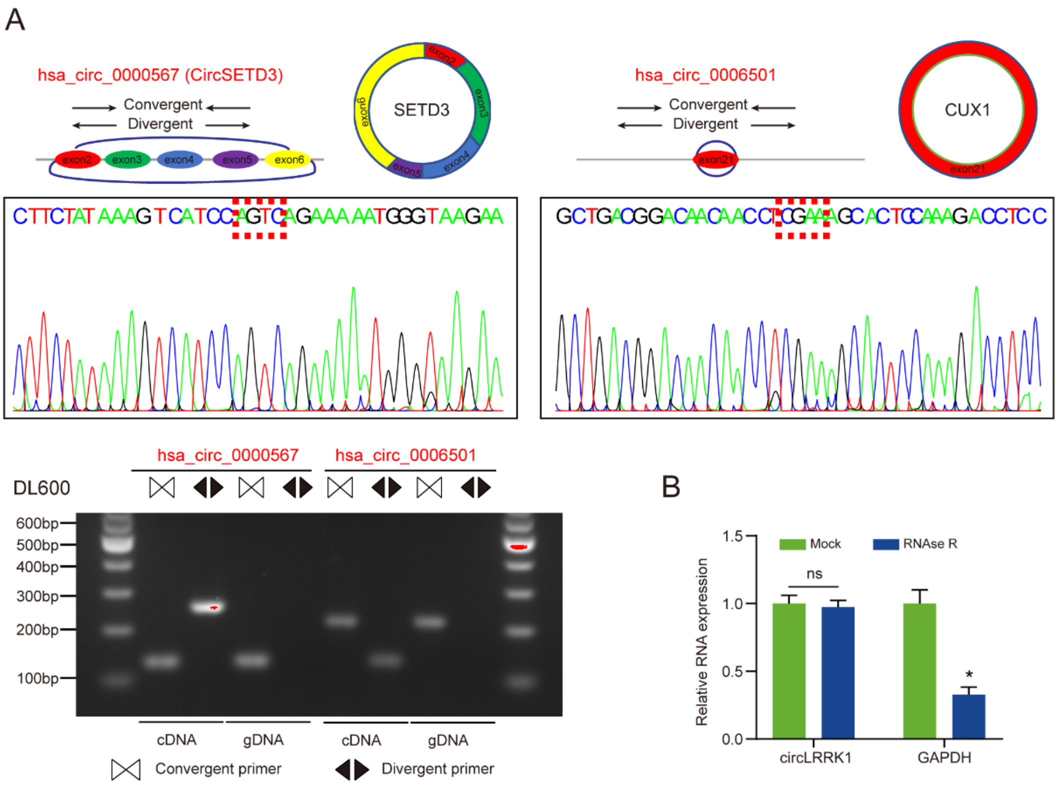

Table S1. All special primers were applied in our study.

Primer	Sequence
MiR-653	Forward: 5’-GTGTTGAAACAATCTCTACTG-3’
	Reverse: 5’-GAACATGTCTGCGTATCTC-3’
U6	Forward: Universal U6 Primer F（human)
	Reverse: Universal PCR Primer R
GAPDH	Forward: 5’ GCATCTTCTTGTGCAGTGCC-3’
	Reverse: 5’ TACGGCCAAATCCGTTCACA-3’
MiR-431	Forward: 5’-GCGGCGGTGTCTTGCAGGCCGTC-3’
	Reverse: 5’-ATCCAGTGCAGGGTCCGAGG-3’
Let-7g	Forward: 5’-ACACTCCAGCTGGGTGAGGTAGTAGTTTGTAC-3’
	Reverse: 5’-CTCAACTGGTGTCGTGGA-3’
MiR-130a	Forward: 5’-GCGCGGATCCAGGCGGCAAAAGGAAGAGTGGTG-3’
	Reverse: 5’-CGGCGAATTCCACAAGCACTGCATACAGAAGTAG-3’
MiR-552	Forward: 5’-CGGCGAATTCCACAAGCACTGCATACAGAAGTAG-3’
	Reverse: 5’-CGAACGCTTCACGAATTTG-3’
KLF6	Forward: 5’ CACACAGGAGAAAAGCCTTACAGAT, 3’
	Reverse: 5’ GTCAGACCTGGAGAAACACCTG-3’

GNAQ	Forward: 5’−ATTGTGTCTTCCCTCC−3’
STYK1	Reverse: 5’−GGTTTCATGGACTCAG−3’
SULT1B1	Forward: 5’-CAGTGGGAAGGAGGGACTGA-3’
	Reverse: 5’-TGCAGCCCAGTGAAATTGGA-3’
MiR-653 promoter	Forward: 5’-CTCGTAATGCCAAGGATGTTTCA-3’
	Reverse: 5’-CTTGATTTCCTCCTTTGGATTCTCT-3’
SOWAHB	Forward: 5'-CATACACTCAGTGGGCTTGA -3'
	Reverse: 5' –TGCCACCAACAGCAGTAGTC-3'
CircSETD3 (Divergent)	Forward: 5'-GGGAGGGCGAGACTCAGACA-3'
	Reverse: 5'-GCGACCGAGTTGACGAAGC-3'
CircSETD3 (Convergent)	Forward: 5'-TTCAAACCCTCCCCAGTGAA-3'
	Reverse: 5'-AGCTCACTGGTCAGGTTCAA-3'
hsa_circ_0006501 (Divergent)	Forward: 5'-AAACTCAGAAATCTGGCACT-3'
	Reverse: 5'-CATACTCTTCCCACTCTTTT-3'
hsa_circ_0006501 (Convergent)	Forward: 5'-TGGACACCTACGGCATAACC-3'
	Reverse: 5'-ACTCACTGGAACTCATCGGG-3
hsa_circ_0070039 (Divergent)	Forward: 5'-CAGTGAGTCGGTGAAGAGCCT -3'
	Reverse: 5'-GCTTGGTTATGCCGTAGGTGT -3
hsa_circ_0002273 (Divergent)	Forward: 5'-TCGAAGCCAACAACAACAGT-3'
	Reverse: 5'-GAGTCCAGTAGTGCCTGTGT-3
hsa_circ_0028255 (Divergent)	Forward: 5'-AGCCTGTTTGTTTTGCACCA-3'
	Reverse: 5'-AGATGGGCTAGGTTGTGCTT-3
	Forward: 5'-AGCCTGTTTGTTTTGCACCA-3'
	Reverse: 5'-ACTAGCAGGCGTAGGAGATG-3

Table S2. The detailed clinical information for dataset TCGA-COAD.

A0_Samples	A18_Sex	A19_Height	A1_OS	A20_Weight	A21_BMI	A22_histological_type	A23_Tumor_tissue_site	A2_Event	A3_T	A4_N	A5_M	A6_Stage
TCGA-AA-A00L	MALE	Not Available	1157	Not Available		Colon Adenocarcinoma	Colon	Alive	T3	N0	M0	Stage IIA
TCGA-DM-A28H	MALE	178	3561	87	27.45865	Colon Adenocarcinoma	Colon	Alive	T3	N2	M0	Stage IIIC
TCGA-AA-	MAL	Not Ava	31	Not Available		Colon Adenocarci	Colon	Alive	T2	N0	M0	Stage

A01V	E	ilable				noma						I
TCGA-AA-3693	FEMALE	Not Available	0	Not Available		Colon Adenocarcinoma	Colon	Alive	T4	N1	M1	Stage IV
TCGA-AA-A01R	MALE	Not Available	1065	Not Available		Colon Mucinous Adenocarcinoma	Colon	Alive	T3	N2	M0	Stage III
TCGA-CM-6675	MALE	187	397	93.9	26.85235	Colon Adenocarcinoma	Colon	Alive	T3	N2b	M1b	Stage IVB
TCGA-A6-6652	MALE	185	751	137.6	40.20453	Colon Adenocarcinoma	Colon	Alive	T3	N0	M1	Stage IVA
TCGA-CM-6166	FEMALE	153	669	45	19.22338	Colon Adenocarcinoma	Colon	Alive	T2	N0	M0	Stage I
TCGA-AA-A01F	MALE	Not Available	974	Not Available		Colon Adenocarcinoma	Colon	Alive	T3	N1	M0	Stage IIIB
TCGA-QG-A5YW	FEMALE	170	896	84.37	29.19377	Colon Mucinous Adenocarcinoma	Colon	Alive	T3	N2b	MX	Stage IIIC
TCGA-DM-A1DB	MALE	167	1348	75	26.89232	Colon Adenocarcinoma	Colon	Dead	T3	N0	M0	Stage IIA
TCGA-AA-3956	MALE	Not Available	1035	Not Available		Colon Adenocarcinoma	Colon	Alive	T3	N0	M0	Stage IIA
TCGA-AA-3833	FEMALE	Not Available	485	Not Available		Colon Adenocarcinoma	Colon	Alive	T3	N0	M0	Stage IIA
TCGA-	FEM	145	884	80.2	38.14	Colon Adenocarci	Colon	Alive	T2	N1a	M0	Stage

CM-5341	ALE				507	noma						IIIA
TCGA-D5-5540	MALE	175	1706	83	27.102204	Colon Adenocarcinoma	Colon	Alive	T3	N0	M0	Stage IIA
TCGA-D5-6898	FEMALE	164	229	100	37.180025	Colon Adenocarcinoma	Colon	Alive	T2	N0	M0	Stage I
TCGA-AA-A02O	MALE	Not Available	28	Not Available		Colon Adenocarcinoma	Colon	Alive	T3	N0	M0	Stage II
TCGA-AA-A00Q	FEMALE	Not Available	1278	Not Available		Colon Adenocarcinoma	Colon	Alive	T4	N1	M0	Stage IIIB
TCGA-AA-3666	MALE	Not Available	61	Not Available		Colon Adenocarcinoma	Colon	Dead	T3	N1	M0	Stage III
TCGA-AZ-6608	FEMALE	Not Available	59	Not Available		Colon Adenocarcinoma	Colon	Dead	T2	N1	M0	Stage IIIA
TCGA-A6-A565	FEMALE	150	494	66.7	29.644444	Colon Mucinous Adenocarcinoma	Colon	Dead	T3	N2	MX	Stage IIIC
TCGA-D5-6537	MALE	176	146	80	25.826645	Colon Adenocarcinoma	Colon	Dead	T3	N1a	MX	Stage IIIB
TCGA-A6-6142	FEMALE	162.5	763	88	33.325444	Colon Adenocarcinoma	Colon	Alive	T3	N1a	M1a	Stage IVA
TCGA-AA-3855	MALE	Not Available	975	Not Available		Colon Adenocarcinoma	Colon	Alive	T2	N0	M0	Stage I
TCGA-NH-	FEM	165.1	389	107	39.25	Colon Mucinous	Colon	Alive	T4b	N1b	M1b	Stage

A6GC	ALE				452	Adenocarcinoma						IVB
TCGA-AA-3814	FEMALE	Not Available	0	Not Available		Colon Adenocarcinoma	Colon	Alive	T3	N0	M0	Stage IIA
TCGA-AA-3667	FEMALE	Not Available	426	Not Available		Colon Adenocarcinoma	Colon	Alive	T2	N0	M0	Stage I
TCGA-G4-6303	FEMALE	Not Available	2003	69		Colon Adenocarcinoma	Colon	Dead	T3	N1	M1	Stage IV
TCGA-AD-6963	MALE	193	834	107	28.7256	Colon Adenocarcinoma	Colon	Alive	T3	N0	MX	
TCGA-AA-3516	FEMALE	Not Available	396	Not Available		Colon Mucinous Adenocarcinoma	Colon	Dead	T3	N2	M0	Stage III
TCGA-CM-6674	MALE	167	394	80.7	28.93614	Colon Adenocarcinoma	Colon	Alive	T3	N0	M0	Stage IIA
TCGA-F4-6461	FEMALE	151	338	57	24.9989	Colon Adenocarcinoma	Colon	Dead	T4b	N2	M0	Stage IIIC
TCGA-AA-3979	MALE	Not Available	730	Not Available		Colon Adenocarcinoma	Colon	Alive	T3	N0	M0	Stage IIA
TCGA-AA-3821	FEMALE	Not Available	31	Not Available		Colon Mucinous Adenocarcinoma	Colon	Alive	T2	N0	M0	Stage I
TCGA-AA-3866	FEMALE	Not Available	518	Not Available		Colon Adenocarcinoma	Colon	Alive	T2	N0	M0	Stage I
TCGA-AA-A00O	FEMAL	Not Availabl	822	Not Available		Colon Adenocarcinoma	Colon	Alive	T3	N2	M0	Stage IIIC

	E	e										
TCGA-F4-6806	FEMALE	172	1260	70	23.661444	Colon Adenocarcinoma	Colon	Alive	T2	N0	M0	Stage I
TCGA-G4-6311	MALE	173.5	1199	69.9	23.220886	Colon Adenocarcinoma	Colon	Alive	T3	N1	MX	Stage III
TCGA-AA-3862	MALE	Not Available	914	Not Available		Colon Adenocarcinoma	Colon	Alive	T3	N0	M0	Stage IIA
TCGA-G4-6309	FEMALE	176	2600	65.4	21.113312	Colon Adenocarcinoma	Colon	Alive	T3	N1	M0	Stage IIIB
TCGA-CK-5913	FEMALE	Not Available	1561	Not Available		Colon Adenocarcinoma	Colon	Alive	T3	N0	MX	Stage IIA
TCGA-A6-5659	MALE	180	926	117.1	36.141198	Colon Adenocarcinoma	Colon	Alive	T2	N0	M0	Stage I
TCGA-AA-3517	MALE	Not Available	1186	Not Available		Colon Adenocarcinoma	Colon	Alive	T3	N0	M0	Stage IIA
TCGA-CA-5796	FEMALE	Not Available	377	Not Available		Colon Mucinous Adenocarcinoma	Colon	Alive	T3	N0	M0	Stage IIA
TCGA-DM-A1D9	FEMALE	153	4270	98	41.864244	Colon Adenocarcinoma	Colon	Alive	T3	N0	M0	Stage IIA
TCGA-D5-6536	MALE	165	543	83	30.486669	Colon Adenocarcinoma	Colon	Alive	T3	N0	M0	Stage IIA
TCGA-AA-A024	MALE	Not Availabl	1188	Not Available		Colon Mucinous Adenocarci	Colon	Dead	T3	N0	M0	Stage II

		e				noma						
TCGA-AA-A00J	MALE	Not Available	549	Not Available		Colon Mucinous Adenocarcinoma	Colon	Alive	T4	N1	M0	Stage IIIB
TCGA-AA-3696	FEMALE	Not Available	153	Not Available		Colon Adenocarcinoma	Colon	Dead	T3	N1	M1	Stage IV
TCGA-F4-6463	MALE	174	1087	62	20.47827	Colon Mucinous Adenocarcinoma	Colon	Alive	T3	N0	M0	Stage IIA
TCGA-CK-6748	FEMALE	Not Available	61	Not Available		Colon Mucinous Adenocarcinoma	Colon	Alive	T3	N1	M1	Stage IV
TCGA-AA-3947	FEMALE	Not Available	1004	Not Available		Colon Mucinous Adenocarcinoma	Colon	Alive	T4	N0	M0	Stage IIB
TCGA-A6-5660	MALE	170	888	94.4	32.66436	Colon Adenocarcinoma	Colon	Alive	T3	N2b	M0	Stage IIIC
TCGA-AA-A03F	FEMALE	Not Available	0	Not Available		Colon Mucinous Adenocarcinoma	Colon	Alive	T3	N2	M0	Stage III
TCGA-DM-A280	FEMALE	159	236	63	24.9199	Colon Mucinous Adenocarcinoma	Colon	Dead	T3	N0	M0	Stage IIA
TCGA-AA-A01I	MALE	Not Available	943	Not Available		Colon Adenocarcinoma	Colon	Alive	T2	N0	M0	Stage I
TCGA-AA-3980	FEMALE	Not Available	242	Not Available		Colon Adenocarcinoma	Colon	Alive	T2	N0	M0	Stage I
TCGA-AA-3675	MALE	Not Availabl	1431	Not Available		Colon Adenocarcinoma	Colon	Alive	T3	N0	M0	Stage II

		e										
TCGA-CK-4947	FEMALE	Not Available	534	Not Available		Colon Adenocarcinoma	Colon	Alive	T4	N1	M0	Stage IIIB
TCGA-AA-A00W	MALE	Not Available	456	Not Available		Colon Adenocarcinoma	Colon	Alive	T1	N0	M0	Stage I
TCGA-AA-3531	FEMALE	Not Available	1035	Not Available		Colon Adenocarcinoma	Colon	Alive	T3	N0	M0	Stage IIA
TCGA-AA-3695	FEMALE	Not Available	0	Not Available		Colon Adenocarcinoma	Colon	Alive	T3	N2	M1	Stage IV
TCGA-NH-A6GB	FEMALE	167.6	476	83	29.548133	Colon Adenocarcinoma	Colon	Alive	T3	N2b	MX	Stage IIIC
TCGA-NH-A8F8	MALE	172.7	511	74	24.811166	Colon Adenocarcinoma	Colon	Dead	T4a	N2b	M1	Stage IV
TCGA-AA-A004	MALE	Not Available	424	Not Available		Colon Adenocarcinoma	Colon	Alive	T3	N0	M0	Stage IIA
TCGA-AA-3994	MALE	Not Available	822	Not Available		Colon Mucinous Adenocarcinoma	Colon	Alive	T3	N1	M0	Stage IIIB
TCGA-DM-A0XF	FEMALE	170	1162	85	29.411176	Discrepancy	Colon	Dead	T3	N2	M0	Stage IIIC
TCGA-AA-3858	MALE	Not Available	945	Not Available		Colon Adenocarcinoma	Colon	Alive	T2	N0	M0	Stage I
TCGA-G4-6586	FEMAL	152.1	1089	63.2	27.3186	Colon Adenocarcinoma	Colon	Alive	T3	N0	M0	Stage IIA

	E				1							
TCGA-QL-A97D	FEMALE	Not Available	666	Not Available		Colon Adenocarcinoma	Colon	Alive	T2	N0	MX	Stage I
TCGA-AZ-6606	MALE	Not Available	357	Not Available		Colon Adenocarcinoma	Colon	Dead	T4	N2	M1	Stage IV
TCGA-CK-6747	FEMALE	Not Available	820	Not Available		Colon Adenocarcinoma	Colon	Alive	T3	N0	MX	Stage IIA
TCGA-CM-4750	FEMALE	173	244	84.5	28.233349	Colon Adenocarcinoma	Colon	Alive	T1	N1b	M0	Stage IIIA
TCGA-A6-3809	FEMALE	160	996	102.1	39.888281	Colon Mucinous Adenocarcinoma	Colon	Alive	T4	N0	M0	Stage IIB
TCGA-AA-3514	FEMALE	Not Available	31	Not Available		Colon Adenocarcinoma	Colon	Alive	T2	N0	M0	Stage I
TCGA-DM-A288	MALE	170	427	85	29.411176	Colon Mucinous Adenocarcinoma	Colon	Dead	T3	N2	M0	Stage IIIC
TCGA-AZ-4684	MALE	Not Available	1977	94		Colon Adenocarcinoma	Colon	Alive	T3	N2	M1	Stage IVA
TCGA-AA-3538	FEMALE	Not Available	791	Not Available		Colon Adenocarcinoma	Colon	Alive	T2	N0	M0	Stage I
TCGA-AA-A01D	FEMALE	Not Available	334	Not Available		Colon Mucinous Adenocarcinoma	Colon	Dead	T3	N2	M0	Stage IIIC
TCGA-4T-AA8	FEMAL	167.6	385	107.956	38.4325	Colon Mucinous Adenocarci	Colon	Alive	T3	N0	MX	Stage IIA

H	E				1	noma						
TCGA-F4-6807	FEMALE	166	1309	57	20.685155	Colon Adenocarcinoma	Colon	Alive	T3	N2b	M0	Stage IIIC
TCGA-AA-3561	MALE	Not Available	424	Not Available		Colon Adenocarcinoma	Colon	Alive	T3	N0	M0	Stage IIA
TCGA-NH-A6GA	MALE	162.6	302	92	34.797474	Colon Adenocarcinoma	Colon	Dead	T4a	N2a	MX	Stage IIIC
TCGA-D5-6535	FEMALE	157	460	65	26.370244	Colon Adenocarcinoma	Colon	Alive	T3	N1	MX	Stage IIIB
TCGA-G4-6295	FEMALE	Not Available	254	59		Colon Adenocarcinoma	Colon	Alive	T3	N0	M0	Stage II
TCGA-A6-2682	MALE	180	424	96.5	29.783955	Colon Adenocarcinoma	Colon	Dead	T4b	N1	M1	Stage IV
TCGA-AD-A5EK	MALE	190	500	100	27.700833	Colon Adenocarcinoma	Colon	Alive	T2	N0	MX	Stage I
TCGA-D5-5541	MALE	170	1701	81	28.027768	Colon Adenocarcinoma	Colon	Alive	T3	N1a	M0	Stage IIIB
TCGA-AA-3713	MALE	Not Available	579	Not Available		Colon Adenocarcinoma	Colon	Alive	T3	N0	M1	Stage IV
TCGA-G4-6320	MALE	193	804	139.7	37.504436	Colon Adenocarcinoma	Colon	Alive	T3	N1	MX	Stage III
TCGA-CA-6715	MALE	158	383	57	22.832888	Colon Adenocarcinoma	Colon	Alive	T3	N1	M0	Stage IIIB

TCGA-AA-3867	MALE	Not Available	731	Not Available		Colon Adenocarcinoma	Colon	Alive	T3	N2	M1	Stage IV
TCGA-A6-2680	FEMALE	151	1068	52.6	23.06916	Colon Adenocarcinoma	Colon	Alive	T3	N0	M0	Stage II
TCGA-AA-3864	MALE	Not Available	1612	Not Available		Colon Adenocarcinoma	Colon	Alive	T3	N0	M0	Stage II
TCGA-AA-3972	MALE	Not Available	1551	Not Available		Colon Adenocarcinoma	Colon	Alive	T3	N1	M1	Stage IV
TCGA-CM-5348	MALE	163	699	85.5	32.18036	Colon Adenocarcinoma	Colon	Alive	T3	N1a	M0	Stage IIIB
TCGA-AY-A8YK	MALE	185	573	86.1	25.15705	Colon Adenocarcinoma	Colon	Alive	T3	N2a	M1	Stage IVA
TCGA-F4-6805	FEMALE	162	1047	60	22.86237	Colon Adenocarcinoma	Colon	Alive	T3	N0	M0	Stage IIA
TCGA-AA-3489	MALE	Not Available	214	Not Available		Colon Adenocarcinoma	Colon	Dead	T3	N0	M0	Stage II
TCGA-A6-2678	FEMALE	171	1286	96.1	32.86481	Colon Adenocarcinoma	Colon	Alive	T3	N1	M0	Stage IIIB
TCGA-DM-A28M	MALE	172	2895	90	30.42185	Colon Adenocarcinoma	Colon	Alive	T3	N0	M0	Stage IIA
TCGA-AA-3848	FEMAL	Not Availabl	306	Not Available		Colon Adenocarcinoma	Colon	Dead	T3	N2	M0	Stage IIIC

	E	e										
TCGA-AA-3519	MALE	Not Available	276	Not Available		Colon Adenocarcinoma	Colon	Alive	T3	N1	M0	Stage III
TCGA-AA-A01X	FEMALE	Not Available	791	Not Available		Colon Adenocarcinoma	Colon	Alive	T2	N1	M0	Stage III
TCGA-AD-6964	MALE	183	331	91	27.1731	Colon Adenocarcinoma	Colon	Dead	T4a	N2b		
TCGA-D5-6928	MALE	174	354	93	30.7174	Colon Mucinous Adenocarcinoma	Colon	Alive	T3	N0	M0	Stage IIA
TCGA-AZ-4323	MALE	Not Available	43	Not Available		Colon Adenocarcinoma	Colon	Dead	T4	N2	M1	Stage IV
TCGA-D5-5539	MALE	168	596	83	29.4076	Colon Mucinous Adenocarcinoma	Colon	Alive	T3	N1	M0	Stage IIIA
TCGA-DM-A1DA	FEMALE	163	228	62	23.33547	Colon Adenocarcinoma	Colon	Dead	T3	N2	M0	Stage IIIC
TCGA-D5-6923	MALE	187	378	109	31.17047	Colon Adenocarcinoma	Colon	Alive	T2	N0	M0	Stage I
TCGA-A6-6781	MALE	190.5	598	87.5	24.111116	Colon Mucinous Adenocarcinoma	Colon	Alive	T4b	N1b	MX	Stage IIIC
TCGA-AA-A02F	FEMALE	Not Available	1216	Not Available		Colon Adenocarcinoma	Colon	Alive	T3	N1	M1	Stage IV
TCGA-DM-	MALE	175		75	24.4898	Discrepancy	Colon		T3	N0	M0	Stage IIA

A1D7												
TCGA-CK-4950	FEMALE	Not Available	2599	Not Available		Colon Mucinous Adenocarcinoma	Colon	Alive	T3	N1	M0	Stage IIIB
TCGA-AA-3973	MALE	Not Available	397	Not Available		Colon Adenocarcinoma	Colon	Alive	T4	N1	M1	Stage IV
TCGA-5M-AATE	MALE	177	1200	75.4	24.067716	Colon Adenocarcinoma	Colon	Alive	T3	N0	M0	Stage IIA
TCGA-A6-2671	MALE	182.8	1331	67.2	20.110022	Colon Adenocarcinoma	Colon	Dead	T3	N2	M1	Stage IV
TCGA-AZ-6599	MALE	Not Available	206	Not Available		Colon Adenocarcinoma	Colon	Dead	T2	N0	MX	Stage I
TCGA-4N-A93T	MALE	167.64	146	134	47.681445	Colon Adenocarcinoma	Colon	Alive	T4a	N1b	M0	Stage IIIB
TCGA-AA-3672	FEMALE	Not Available	0	Not Available		Colon Adenocarcinoma	Colon	Alive	T3	N1	M0	Stage III
TCGA-DM-A1D6	MALE	173	570	80	26.729993	Colon Mucinous Adenocarcinoma	Colon	Dead	T3	N0	M0	Stage IIA
TCGA-AA-3977	MALE	Not Available	761	Not Available		Colon Adenocarcinoma	Colon	Alive	T2	N0		
TCGA-AA-3877	FEMALE	Not Available	943	Not Available		Colon Mucinous Adenocarcinoma	Colon	Alive	T1	N0	M0	Stage I
TCGA-AA-3530	MALE	Not Availabl	580	Not Available		Colon Adenocarcinoma	Colon	Alive	T2	N0	M0	Stage I

		e										
TCGA-A6-3807	FEMALE	163	1054	109.5	41.213344	Colon Adenocarcinoma	Colon	Alive	T3	N2	M0	Stage IIIC
TCGA-AA-3845	FEMALE	Not Available	0	Not Available		Colon Adenocarcinoma	Colon	Dead	T3	N0	M0	Stage IIA
TCGA-AZ-6607	MALE	Not Available	97	Not Available		Colon Adenocarcinoma	Colon	Dead	T4	N2	M1	Stage IV
TCGA-DM-A1D8	FEMALE	158	383	65	26.037749	Colon Adenocarcinoma	Colon	Dead	T3	N1		
TCGA-CA-6717	MALE	168	388	58	20.549989	Colon Mucinous Adenocarcinoma	Colon	Alive	T3	N0	M0	Stage IIA
TCGA-G4-6299	MALE	130	2268	88	52.071101	Colon Adenocarcinoma	Colon	Alive	T3	N2	M0	Stage IIIC
TCGA-G4-6626	MALE	Not Available	1	59		Colon Adenocarcinoma	Colon	Dead	T3	N0	M0	Stage IIA
TCGA-A6-6140	MALE	177.8	734	78.9	24.958821	Colon Adenocarcinoma	Colon	Alive	T3	N0	M0	Stage IIA
TCGA-AA-3506	MALE	Not Available	1765	Not Available		Colon Adenocarcinoma	Colon	Alive	T2	N0	M0	Stage I
TCGA-AA-3970	MALE	Not Available	1096	Not Available		Colon Adenocarcinoma	Colon	Alive	T3	N0	M0	Stage IIA
TCGA-A6-5666	MALE	193	995	95.4	25.6114	Colon Adenocarcinoma	Colon	Alive	T4b	N0	M0	Stage IIC

					3							
TCGA-AA-3679	MALE	Not Available	457	Not Available		Colon Adenocarcinoma	Colon	Alive	T3	N2	M1	Stage IV
TCGA-AA-3548	FEMALE	Not Available	1034	Not Available		Colon Adenocarcinoma	Colon	Alive	T3	N2	M0	Stage IIIC
TCGA-AA-A017	FEMALE	Not Available	457	Not Available		Colon Adenocarcinoma	Colon	Alive	T3	N0	M0	Stage IIA
TCGA-A6-6648	MALE	182.8	766	87	26.035556	Colon Adenocarcinoma	Colon	Alive	T3	N0	M1a	Stage IVA
TCGA-AA-3949	FEMALE	Not Available	791	Not Available		Colon Mucinous Adenocarcinoma	Colon	Alive	T3	N1	M0	Stage IIIB
TCGA-F4-6569	MALE	180	1087	60	18.51852	Colon Adenocarcinoma	Colon	Alive	T2	N0	M0	Stage I
TCGA-AA-A00Z	MALE	Not Available	669	Not Available		Colon Adenocarcinoma	Colon	Alive	T3	N0	M0	Stage IIA
TCGA-NH-A50U	MALE	172.7	334	88.5	29.6728	Colon Mucinous Adenocarcinoma	Colon	Dead	T4a	N0	M1a	Stage IVA
TCGA-AA-3510	MALE	Not Available	1946	Not Available		Colon Adenocarcinoma	Colon	Alive	T3	N0	M0	Stage II
TCGA-AA-3552	MALE	Not Available	396	Not Available		Colon Adenocarcinoma	Colon	Dead	T3	N2	M0	Stage IIIC
TCGA-CK-5916	FEMAL	Not Availabl	643	Not Available		Colon Adenocarcinoma	Colon	Dead	T1	N0	M0	Stage I

	E	e										
TCGA-CK-6746	FEMALE	Not Available	0	Not Available		Colon Adenocarcinoma	Colon	Alive	T4b	N0	MX	Stage IIB
TCGA-AA-3543	MALE	Not Available	30	Not Available		Colon Mucinous Adenocarcinoma	Colon	Alive	T2	N0	M0	Stage I
TCGA-CM-5864	MALE	180	457	103	31.790012	Colon Adenocarcinoma	Colon	Alive	T2	N0	M0	Stage I
TCGA-CM-4747	MALE	188	761	76.7	21.701	Colon Adenocarcinoma	Colon	Alive	T4a	N1b	M1a	Stage IVA
TCGA-AA-3655	MALE	Not Available	1856	Not Available		Colon Adenocarcinoma	Colon	Alive	T3	N0	M0	Stage II
TCGA-G4-6306	MALE	170	1359	110	38.062228	Colon Adenocarcinoma	Colon	Alive	T2	N0	M0	
TCGA-AY-4070	FEMALE	160	496	100.8	39.375	Colon Adenocarcinoma	Colon	Dead	T3	N2	M0	Stage IIIC
TCGA-AA-3976	MALE	Not Available	791	Not Available		Colon Adenocarcinoma	Colon	Alive	T2	N1	M0	Stage IIIA
TCGA-CM-6171	FEMALE	162	427	71.2	27.130001	Colon Adenocarcinoma	Colon	Alive	T2	N0	M0	Stage I
TCGA-CM-4751	MALE	183	822	115	34.339963	Colon Adenocarcinoma	Colon	Alive	T3	N1b	M0	Stage IIIB
TCGA-AA-3856	MALE	Not Availabl	30	Not Available		Colon Adenocarcinoma	Colon	Alive	T3	N0	M0	Stage IIA

		e										
TCGA-AZ-4614	FEMALE	Not Available	172	70		Colon Adenocarcinoma	Colon	Dead	T4a	N1	M1	Stage IVA
TCGA-CM-6170	FEMALE	145	457	85.8	40.80856	Colon Adenocarcinoma	Colon	Alive	T2	N0	M0	Stage I
TCGA-D5-6924	MALE	165	435	85	31.2213	Colon Adenocarcinoma	Colon	Alive	T3	N0	M0	Stage IIA
TCGA-CM-5349	FEMALE	156	915	54.4	22.35371	Colon Adenocarcinoma	Colon	Alive	T3	N0	M0	Stage IIA
TCGA-AZ-4682	MALE	Not Available	680	98		Colon Adenocarcinoma	Colon	Dead	T3	N0	M1	Stage IVA
TCGA-AA-3560	FEMALE	Not Available	608	Not Available		Colon Adenocarcinoma	Colon	Alive	T3	N2	M0	Stage IIIC
TCGA-D5-7000	FEMALE	154	312	60	25.29938	Colon Mucinous Adenocarcinoma	Colon	Alive	T2	N0	M0	Stage I
TCGA-CM-6172	FEMALE	151	335	77.6	34.0336	Colon Adenocarcinoma	Colon	Alive	T3	N1a	M0	Stage IIIB
TCGA-D5-6532	MALE	174	555	92	30.38711	Colon Adenocarcinoma	Colon	Alive	T3	N0	M0	Stage IIA
TCGA-5M-AAT4	MALE	Not Available	49	Not Available		Colon Adenocarcinoma	Colon	Dead	T3	N0	M1b	Stage IV
TCGA-CA-5255	MALE	172	376	56	18.92915	Colon Adenocarcinoma	Colon	Alive	T3	N0	M0	Stage IIA

TCGA-A6-2676	FEMALE	154.9	1305	45.9	19.129977	Colon Adenocarcinoma	Colon	Dead	T4	N0	M0	Stage IIB
TCGA-A6-5656	MALE	179	1001	76.6	23.906687	Colon Adenocarcinoma	Colon	Alive	T2	N0	M0	Stage I
TCGA-F4-6809	FEMALE	150	403	88	39.111111	Colon Adenocarcinoma	Colon	Dead	T3	N1	M1	Stage IVA
TCGA-AA-3715	MALE	Not Available	579	Not Available		Colon Adenocarcinoma	Colon	Dead	T3	N0	M0	Stage II
TCGA-F4-6808	FEMALE	166	1024	70	25.402282	Colon Adenocarcinoma	Colon	Alive	T1	N0	M0	Stage I
TCGA-AY-6386	FEMALE	153	542	86	36.737801	Colon Adenocarcinoma	Colon	Alive	T3	N1a	M0	Stage IIIB
TCGA-CK-4952	FEMALE	Not Available	475	Not Available		Colon Mucinous Adenocarcinoma	Colon	Alive	T4	N2	M0	Stage IIIC
TCGA-AA-3831	MALE	Not Available	547	Not Available		Colon Adenocarcinoma	Colon	Alive	T3	N0	M0	Stage IIA
TCGA-AA-A010	FEMALE	Not Available	1064	Not Available		Colon Adenocarcinoma	Colon	Alive	T4	N0	M0	Stage IIB
TCGA-AD-6901	MALE	170	682	69	23.875543	Colon Adenocarcinoma	Colon	Dead	T3	N0	MX	
TCGA-D5-6929	FEMALE	167	408	80	28.685514	Colon Adenocarcinoma	Colon	Alive	T3	N1	M1	Stage IV

TCGA-AA-3673	FEMALE	Not Available	1522	Not Available		Colon Adenocarcinoma	Colon	Alive	T3	N0	M0	Stage II
TCGA-AA-A01K	FEMALE	Not Available	943	Not Available		Colon Adenocarcinoma	Colon	Alive	T3	N2	M0	Stage IIIC
TCGA-G4-6307	FEMALE	166	1674	71	25.76571	Colon Adenocarcinoma	Colon	Alive	T3	N1	M0	Stage IIIB
TCGA-A6-2685	FEMALE	171	1133	77.3	26.43548	Colon Adenocarcinoma	Colon	Alive	T3	N0	M0	Stage IIA
TCGA-AA-3556	MALE	Not Available	700	Not Available		Not Available	Colon	Alive	T2	N0	M0	Stage I
TCGA-AZ-4313	FEMALE	Not Available	2310	57		Colon Adenocarcinoma	Colon	Alive	T1	N0	M0	Stage I
TCGA-D5-5538	FEMALE	165	1661	70	25.71166	Colon Adenocarcinoma	Colon	Dead	T3	N1b	M0	Stage IIIB
TCGA-5M-AAT6	FEMALE	162	290	99.1	37.76101	Colon Adenocarcinoma	Colon	Dead	T4a	N2b	M1a	Stage IV
TCGA-G4-6310	MALE	173	1935	102.5	34.24772	Colon Adenocarcinoma	Colon	Alive	T3	N1	M0	Stage IIIB
TCGA-AM-5820	FEMALE	164	14	98	36.43664	Colon Adenocarcinoma	Colon	Alive	T4a	N2	M1	Stage IVA
TCGA-QG-A5Z1	MALE	Not Available	256	91.1		Colon Adenocarcinoma	Colon	Dead	T3	N1b	MX	Stage IIIB

TCGA-D5-6541	MALE	174	474	85	28.07504	Colon Adenocarcinoma	Colon	Alive	T3	N0	M0	Stage IIA
TCGA-AA-A02J	FEMALE	Not Available	153	Not Available		Colon Adenocarcinoma	Colon	Dead	T3	N0	M1	Stage IV
TCGA-DM-A28A	MALE	172	805	72	24.33748	Colon Adenocarcinoma	Colon	Dead	T3	N2	M0	Stage IIIC
TCGA-AA-3502	MALE	Not Available	1065	Not Available		Colon Adenocarcinoma	Colon	Alive	T2	N0	M0	Stage I
TCGA-A6-5664	MALE	180.3	672	70.3	21.62539	Colon Adenocarcinoma	Colon	Alive	T4a	N2a	MX	Stage IIIC
TCGA-A6-2677	FEMALE	160	740	55.2	21.5625	Colon Adenocarcinoma	Colon	Dead	T3	N2	M0	Stage IIIC
TCGA-AZ-5403	MALE	Not Available	1910	Not Available		Colon Adenocarcinoma	Colon	Dead	T3	N0	MX	Stage II
TCGA-A6-6138	MALE	185.4	685	90.7	26.38687	Colon Adenocarcinoma	Colon	Alive	T2	N0	M0	Stage I
TCGA-AA-3710	FEMALE	Not Available	821	Not Available		Colon Adenocarcinoma	Colon	Alive	T3	N0	M0	Stage IIA
TCGA-AA-A01S	FEMALE	Not Available	31	Not Available		Colon Adenocarcinoma	Colon	Alive	T3	N1	M0	Stage III
TCGA-CA-5256	FEMALE	160	379	52	20.3125	Colon Adenocarcinoma	Colon	Alive	T3	N0	M0	Stage IIA

TCGA-CA-6719	MALE	172	435	61	20.61925	Colon Adenocarcinoma	Colon	Alive	T3	N0	M0	Stage IIA
TCGA-CK-4951	FEMALE	Not Available	1492	Not Available		Colon Mucinous Adenocarcinoma	Colon	Alive	T3	N0	M0	Stage IIA
TCGA-CM-4743	MALE	167	701	84.4	30.26283	Colon Adenocarcinoma	Colon	Alive	T3	N0	M0	Stage IIA
TCGA-AA-3930	MALE	Not Available	61	Not Available		Colon Adenocarcinoma	Colon	Dead	T3	N2	M1	Stage IV
TCGA-AD-6895	MALE	170	763	68	23.52941	Colon Adenocarcinoma	Colon	Alive	T3	N1a	M0	Stage IIIB
TCGA-D5-6540	MALE	186	491	86	24.85837	Colon Mucinous Adenocarcinoma	Colon	Alive	T2	N0	M0	Stage I
TCGA-CA-5797	MALE	Not Available	383	Not Available		Colon Adenocarcinoma	Colon	Alive	T3	N0	M0	Stage IIA
TCGA-AA-A00N	MALE	Not Available	122	Not Available		Colon Mucinous Adenocarcinoma	Colon	Dead	T4	N0	M0	Stage IIB
TCGA-AA-A02E	FEMALE	Not Available	90	Not Available		Colon Adenocarcinoma	Colon	Dead	T3	N1	M1	Stage IV
TCGA-D5-6529	MALE	176	614	106	34.22004	Colon Adenocarcinoma	Colon	Alive	T3	N0	M0	Stage IIA
TCGA-AA-A00R	FEMALE	Not Available	30	Not Available		Colon Adenocarcinoma	Colon	Alive	T2	N0	M0	Stage I

TCGA-G4-6588	FEMALE	166	796	57	20.685155	Colon Adenocarcinoma	Colon	Alive	T3	N0	M0	Stage IIA
TCGA-F4-6460	FEMALE	168	972	73	25.864451	Colon Adenocarcinoma	Colon	Dead	T3	N1	M0	Stage IIIB
TCGA-G4-6304	FEMALE	Not Available	1631	57		Colon Adenocarcinoma	Colon	Alive	T4	N0	M0	Stage IIB
TCGA-AA-3850	MALE	Not Available	0	Not Available		Colon Adenocarcinoma	Colon	Dead	T2	N0	M0	Stage I
TCGA-AA-3558	MALE	Not Available	638	Not Available		Colon Adenocarcinoma	Colon	Alive	T2	N0	M0	Stage I
TCGA-CM-5861	FEMALE	164	457	122	45.3599	Colon Adenocarcinoma	Colon	Alive	T3	N0	M0	Stage IIA
TCGA-G4-6298	MALE	Not Available	0	59.4		Colon Adenocarcinoma	Colon	Dead	T4a	N1	MX	Stage IIIB
TCGA-G4-6315	MALE	170	1883	58	20.0692	Colon Adenocarcinoma	Colon	Alive	T3	N1	M1	Stage IV
TCGA-AA-3841	MALE	Not Available	1124	Not Available		Colon Adenocarcinoma	Colon	Alive	T3	N0	M0	Stage IIA
TCGA-AA-A00U	MALE	Not Available	518	Not Available		Colon Adenocarcinoma	Colon	Alive	T3	N1	M0	Stage IIIB
TCGA-A6-2679	FEMALE	165.1	1366	65.9	24.17638	Colon Adenocarcinoma	Colon	Alive	T3	N0		Stage IIB
TCGA	M	Not	66	Not		Colon	Colon	Ali	T3	N1	MX	Sta

-CK-5914	ALE	Available	9	Available		Adenocarcinoma		ve				ge IIIB
TCGA-DM-A28C	MALE	180	2475	70	21.604944	Colon Adenocarcinoma	Colon	Dead	T3	N0	M0	Stage IIA
TCGA-QG-A5Z2	MALE	183	952	97.5	29.114044	Colon Adenocarcinoma	Colon	Alive	T2	N0	M0	Stage I
TCGA-CM-6168	FEMALE	149	395	52.5	23.647558	Colon Adenocarcinoma	Colon	Alive	T3	N0	M0	Stage IIA
TCGA-A6-2686	FEMALE	155	1126	61.4	25.556671	Colon Adenocarcinoma	Colon	Dead	T3	N0	M0	Stage IIA
TCGA-AA-3544	MALE	Not Available	426	Not Available		Colon Adenocarcinoma	Colon	Alive	T2	N0	M0	Stage I
TCGA-AA-A00D	MALE	Not Available	578	Not Available		Colon Adenocarcinoma	Colon	Alive	T2	N0	M0	Stage I
TCGA-A6-5657	MALE	172	962	99.6	33.666685	Colon Adenocarcinoma	Colon	Alive	T3	N1	M0	Stage IIIB
TCGA-CM-5863	FEMALE	150	457	103.4	45.955556	Colon Mucinous Adenocarcinoma	Colon	Alive	T3	N1b	M0	Stage IIIB
TCGA-AA-3488	MALE	Not Available	153	Not Available		Colon Adenocarcinoma	Colon	Dead	T3	N2	M1	Stage IV
TCGA-AD-6899	MALE	175	176	62	20.2449	Colon Mucinous Adenocarcinoma	Colon	Dead	T4a	N2b	MX	Stage IIIC
TCGA	M	170	18	90	31.	Colon	Colon	De	T3	N0	M0	Sta

-DM-A28G	ALE		49		14187	Adenocarcinoma		ad				ge IIA
TCGA-AA-3562	MALE	Not Available	608	Not Available		Colon Adenocarcinoma	Colon	Alive	T3	N2	M0	Stage IIIC
TCGA-CM-4748	MALE	176	792	89.8	28.99019	Colon Mucinous Adenocarcinoma	Colon	Alive	T4a	N1a	M0	Stage IIIB
TCGA-AA-3968	FEMALE	Not Available	669	Not Available		Colon Adenocarcinoma	Colon	Alive	T2	N0	M0	Stage I
TCGA-G4-6297	FEMALE	Not Available	2506	64		Colon Adenocarcinoma	Colon	Alive	T3	N2	M1	Stage IV
TCGA-CM-5862	MALE	162.5	153	77.6	29.38698	Colon Adenocarcinoma	Colon	Dead	T3	N1a	M1a	Stage IVA
TCGA-CA-6716	MALE	167	371	58	20.79673	Colon Adenocarcinoma	Colon	Alive	T3	N0	M0	Stage IIA
TCGA-AA-A01C	MALE	Not Available	457	Not Available		Colon Adenocarcinoma	Colon	Alive	T2	N1	M0	Stage IIIA
TCGA-AA-3553	FEMALE	Not Available	730	Not Available		Colon Adenocarcinoma	Colon	Alive	T2	N0	M0	Stage I
TCGA-A6-2672	FEMALE	157	1419	51.5	20.89334	Colon Adenocarcinoma	Colon	Alive	T3	N1	M0	Stage IIIB
TCGA-AA-A01Z	MALE	Not Available	1126	Not Available		Colon Adenocarcinoma	Colon	Alive	T3	N0	M0	Stage II
TCGA	FE	150	88	59	26.	Colon	Colon	Ali	T3	N0	M0	Sta

-CM-6164	MALE		3		22222	Adenocarcinoma		ve				ge IIA
TCGA-CK-5912	MALE	Not Available	1493	Not Available		Colon Adenocarcinoma	Colon	Dead	T2	N0	MX	Stage I
TCGA-A6-2675	MALE	160	1321	71.3	27.85156	Colon Adenocarcinoma	Colon	Alive	T3	N0	MX	Stage IIA
TCGA-CM-6678	FEMALE	157	335	59	23.93606	Colon Adenocarcinoma	Colon	Alive	T4a	N1c	M1a	Stage IVA
TCGA-DM-A0X9	FEMALE	170	3641	86	29.75779	Colon Adenocarcinoma	Colon	Alive	T3	N0	M0	Stage IIA
TCGA-F4-6703	MALE	172	1456	84	28.39373	Colon Adenocarcinoma	Colon	Alive	T3	N0	M0	Stage IIA
TCGA-AA-3520	FEMALE	Not Available	731	Not Available		Colon Adenocarcinoma	Colon	Alive	T3	N0	M0	Stage II
TCGA-AD-6888	MALE	183	472	96	28.66613	Colon Adenocarcinoma	Colon	Dead	T3	N1b	M0	Stage IIIB
TCGA-AA-3846	FEMALE	Not Available	518	Not Available		Colon Adenocarcinoma	Colon	Alive	T3	N0	M0	Stage IIA
TCGA-CM-6680	FEMALE	150	366	72.4	32.17778	Colon Adenocarcinoma	Colon	Alive	T3	N2a	M0	Stage IIIB
TCGA-DM-A1HB	MALE	168	4126	70	24.80159	Discrepancy	Colon	Alive	T3	N1	M0	Stage IIIB
TCGA	FE	Not	10	61.3		Colon	Colon	Ali	T3	N1	M1	Sta

-AY-5543	MALE	Available	04			Adenocarcinoma		ve		a		ge IVA
TCGA-G4-6323	MALE	171.5	419	88.4	30.0555	Colon Adenocarcinoma	Colon	Alive	Tis	N0	MX	Stage IA
TCGA-AA-A029	MALE	Not Available	1581	Not Available		Colon Adenocarcinoma	Colon	Alive	T3	N0	M0	Stage II
TCGA-AA-3697	MALE	Not Available	2587	Not Available		Colon Adenocarcinoma	Colon	Alive	T3	N0	M0	Stage II
TCGA-CM-5860	MALE	182	974	100.7	30.40092	Colon Adenocarcinoma	Colon	Alive	T3	N0	M0	Stage IIA
TCGA-AD-5900	MALE	Not Available	370	Not Available		Colon Mucinous Adenocarcinoma	Colon	Alive	T2	N0	MX	Stage I
TCGA-AA-A022	FEMALE	Not Available	0	Not Available		Colon Adenocarcinoma	Colon	Alive	T4	N0	M0	Stage II
TCGA-AA-3875	FEMALE	Not Available	549	Not Available		Colon Adenocarcinoma	Colon	Alive	T1	N0	M0	Stage I
TCGA-AA-3869	MALE	Not Available	822	Not Available		Colon Adenocarcinoma	Colon	Dead	T4	N2	M1	Stage IV
TCGA-AA-3812	FEMALE	Not Available	1066	Not Available		Colon Adenocarcinoma	Colon	Alive	T3	N0	M0	Stage IIA
TCGA-G4-6321	FEMALE	178.2	672	100	31.49087	Colon Adenocarcinoma	Colon	Alive	T2	N1	MX	Stage III
TCGA-	MAL	171	2821	59	20.17	Colon Adenocarci	Colon	Dead	T3	N0	M0	Stage

DM-A1D4	E				715	noma						IIA
TCGA-T9-A92H	MALE	180	362	63.9	19.727222	Colon Adenocarcinoma	Colon	Alive	T3	N0	M0	Stage IIA
TCGA-G4-6302	FEMALE	152.5	0	57	24.509954	Colon Mucinous Adenocarcinoma	Colon	Alive	T3	N0	M0	Stage IIA
TCGA-AA-3542	MALE	Not Available	395	Not Available		Colon Adenocarcinoma	Colon	Alive	T3	N2	M0	Stage IIIC
TCGA-AY-6196	MALE	Not Available	6	Not Available		Colon Mucinous Adenocarcinoma	Colon	Alive	T3	N2b		Stage IIIC
TCGA-A6-6654	FEMALE	173	726	97.4	32.543369	Colon Adenocarcinoma	Colon	Alive	T3	N1	M0	Stage IIIB
TCGA-A6-6653	MALE	182.8	742	98.3	29.417199	Colon Adenocarcinoma	Colon	Alive	T2	N0	M0	Stage I
TCGA-D5-6922	MALE	175	308	83	27.102204	Colon Adenocarcinoma	Colon	Alive	T3	N1	M0	Stage IIIA
TCGA-AA-3861	MALE	Not Available	914	Not Available		Colon Adenocarcinoma	Colon	Alive	T3	N0	M0	Stage IIA
TCGA-CK-4948	FEMALE	Not Available	4502	Not Available		Colon Adenocarcinoma	Colon	Alive	T3	N1	M0	Stage III
TCGA-AA-3986	MALE	Not Available	580	Not Available		Colon Adenocarcinoma	Colon	Alive	T2	N0	M0	Stage I
TCGA-D5-	FEM	152	521	57	24.67	Colon Adenocarci	Colon	Alive	T3	N2	M0	Stage

6538	ALE				105	noma						IIIB
TCGA-AA-3971	MALE	Not Available	489	Not Available		Colon Adenocarcinoma	Colon	Alive	T3	N1	M0	Stage III
TCGA-G4-6314	FEMALE	158.5	1093	84.1	33.4763	Colon Adenocarcinoma	Colon	Alive	T3	N2	M1	Stage IV
TCGA-D5-6533	FEMALE	156	775	51	20.95661	Colon Adenocarcinoma	Colon	Alive	T4b	N0	M0	
TCGA-D5-6926	MALE	175	275	82	26.77551	Colon Adenocarcinoma	Colon	Alive	T4a	N1	M0	Stage IIIB
TCGA-AA-3534	FEMALE	Not Available	882	Not Available		Colon Adenocarcinoma	Colon	Alive	T3	N0	M0	Stage IIA
TCGA-A6-4107	FEMALE	154	987	91.8	38.70805	Colon Adenocarcinoma	Colon	Alive	T3	N1b	M0	Stage IIIB
TCGA-DM-A1D0	FEMALE	170	3974	65	22.49135	Colon Adenocarcinoma	Colon	Alive	T3	N0	M0	Stage IIA
TCGA-AA-3509	FEMALE	Not Available	1915	Not Available		Colon Adenocarcinoma	Colon	Alive	T3	N0	M0	Stage II
TCGA-AA-3684	FEMALE	Not Available	0	Not Available		Colon Mucinous Adenocarcinoma	Colon	Alive	T4	N2	M1	Stage IV
TCGA-AA-3663	MALE	Not Available	212	Not Available		Colon Adenocarcinoma	Colon	Alive	T3	N0	M0	Stage II
TCGA-A6-	MAL	180.3	1014	84.5	25.99	Colon Adenocarci	Colon	Alive	T3	N0	M0	Stage

3808	E				353	noma						IIA
TCGA-AA-3950	FEMALE	Not Available	730	Not Available		Colon Mucinous Adenocarcinoma	Colon	Alive	T3	N0	M0	Stage IIA
TCGA-AA-3494	MALE	Not Available	31	Not Available		Colon Adenocarcinoma	Colon	Alive	T3	N0	M1	Stage IV
TCGA-AA-3854	FEMALE	Not Available	1096	Not Available		Colon Mucinous Adenocarcinoma	Colon	Alive	T2	N0	M0	Stage I
TCGA-AD-6890	MALE	Not Available	746	Not Available		Colon Adenocarcinoma	Colon	Alive	T1	N0	MX	
TCGA-AA-3966	FEMALE	Not Available	61	Not Available		Colon Mucinous Adenocarcinoma	Colon	Alive	T3	N0	M0	Stage IIA
TCGA-AA-A00E	MALE	Not Available	913	Not Available		Colon Adenocarcinoma	Colon	Alive	T3	N0	M0	Stage IIA
TCGA-AA-3982	MALE	Not Available	822	Not Available		Colon Adenocarcinoma	Colon	Alive	T3	N1	M0	Stage IIIB
TCGA-A6-2681	FEMALE	170.1	1387	79.6	27.51088	Colon Adenocarcinoma	Colon	Alive	T3	N0	M0	Stage IIA
TCGA-A6-6649	MALE	190	735	106	29.362888	Colon Adenocarcinoma	Colon	Alive	T3	N1b	M0	Stage IIIB
TCGA-A6-2674	MALE	175.2	1331	85.6	27.887244	Colon Mucinous Adenocarcinoma	Colon	Alive	T3	N2	M1	Stage IV
TCGA-F4-	MAL	183	47	163	48.67	Colon Mucinous	Colon	Alive	T3	N2b	MX	Stage

6704	E				27	Adenocarcinoma						IIIC
TCGA-AA-A00F	MALE	Not Available	1035	Not Available		Colon Adenocarcinoma	Colon	Alive	T3	N2	M0	Stage IIIC
TCGA-AD-A5EJ	FEMALE	163	0	71	26.72287	Colon Adenocarcinoma	Colon	Alive	T3	N0	MX	Stage IIA
TCGA-F4-6570	FEMALE	159	188	59	23.33768	Colon Adenocarcinoma	Colon	Dead	T3	N0	M0	Stage IIA
TCGA-AA-3518	FEMALE	Not Available	31	Not Available		Colon Adenocarcinoma	Colon	Alive	T3	N0	M0	Stage IIA
TCGA-A6-A567	MALE	188	1881	84	23.76641	Colon Adenocarcinoma	Colon	Dead	T3	N1	M1	Stage IV
TCGA-A6-A566	FEMALE	160	758	66.1	25.82031	Colon Mucinous Adenocarcinoma	Colon	Dead	T4	N1	M0	Stage IIIB
TCGA-AA-3529	FEMALE	Not Available	0	Not Available		Colon Adenocarcinoma	Colon	Dead	T3	N2	M0	Stage IIIC
TCGA-AA-A01G	MALE	Not Available	365	Not Available		Colon Mucinous Adenocarcinoma	Colon	Alive	T3	N0	M0	Stage IIA
TCGA-CA-6718	MALE	168	306	63	22.32143	Colon Adenocarcinoma	Colon	Dead	T3	N0	M0	Stage IIA
TCGA-AA-3521	MALE	Not Available		Not Available		Colon Adenocarcinoma	Colon		T3	N0	M0	Stage II
TCGA-CM-	MALE	165	337	100	36.7309	Colon Adenocarcinoma	Colon	Alive	T2	N0	M0	Stage I

6676					5							
TCGA-CM-6165	MALE	189	488	100.7	28.1907	Colon Adenocarcinoma	Colon	Alive	T3	N0	M0	Stage IIA
TCGA-DM-A282	FEMALE	153	4233	44	18.79619	Colon Adenocarcinoma	Colon	Alive	T3	N0	M0	Stage IIA
TCGA-AA-3681	FEMALE	Not Available	182	Not Available		Colon Adenocarcinoma	Colon	Alive	T3	N1	M0	Stage III
TCGA-SS-A7HO	FEMALE	167	1829	97.5	34.96002	Colon Adenocarcinoma	Colon	Alive	T4a	N0	M0	Stage IIB
TCGA-G4-6627	MALE	176	2175	80	25.82645	Colon Adenocarcinoma	Colon	Alive	T3	N0	M0	Stage IIA
TCGA-AA-3549	MALE	Not Available	639	Not Available		Colon Adenocarcinoma	Colon	Alive	T2	N0	M0	Stage I
TCGA-AA-3941	FEMALE	Not Available	730	Not Available		Colon Adenocarcinoma	Colon	Alive	T4	N1b	M1a	Stage IVA
TCGA-AY-6197	MALE	Not Available	652	158		Colon Adenocarcinoma	Colon	Alive	T3	N0		Stage IIA
TCGA-A6-2670	MALE	175.3	775	90.9	29.58013	Colon Adenocarcinoma	Colon	Alive	T3	N0	M0	Stage IIA
TCGA-AA-A01Q	FEMALE	Not Available	31	Not Available		Colon Adenocarcinoma	Colon	Alive	T3	N0	M0	Stage II
TCGA-AA-3678	FEMAL	Not Availabl	1430	Not Available		Colon Adenocarcinoma	Colon	Alive	T2	N1	M0	Stage III

	E	e										
TCGA-D5-5537	MALE	168	1381	75	26.575313	Colon Adenocarcinoma	Colon	Dead	T3	N2	MX	
TCGA-NH-A50T	FEMALE	160.02	553	73.5	28.703376	Colon Adenocarcinoma	Colon	Alive	T3	N0	MX	Stage IIA
TCGA-G4-6322	MALE	182	792	90.9	27.442234	Colon Mucinous Adenocarcinoma	Colon	Alive	T3	N1	MX	Stage IIIB
TCGA-AA-A03J	FEMALE	Not Available	1246	Not Available		Colon Adenocarcinoma	Colon	Alive	T2	N0	M0	Stage I
TCGA-AA-3526	MALE	Not Available	580	Not Available		Colon Adenocarcinoma	Colon	Alive	T2	N0	M0	Stage I
TCGA-A6-2683	FEMALE	166.3	504	57.5	20.791138	Colon Adenocarcinoma	Colon	Dead	T4	N0	M1	Stage IV
TCGA-A6-4105	MALE	180.3	442	83.3	25.624439	Colon Adenocarcinoma	Colon	Dead	T3	N0	M0	Stage IIA
TCGA-A6-6141	MALE	177.8	255	96.8	30.620047	Colon Adenocarcinoma	Colon	Alive	T3	N0	M0	Stage IIA
TCGA-CM-4746	MALE	175	1126	98	32	Colon Adenocarcinoma	Colon	Alive	T2	N0	M0	Stage I
TCGA-AA-3555	FEMALE	Not Available	911	Not Available		Colon Mucinous Adenocarcinoma	Colon	Alive	T3	N0	M0	Stage IIA
TCGA-AD-6889	MALE	Not Availabl	2532	Not Available		Colon Adenocarcinoma	Colon	Dead	T3	N0	M0	Stage IIA

		e										
TCGA-AZ-6600	MALE	Not Available	368	Not Available		Colon Adenocarcinoma	Colon	Dead	T4	N1	M1	Stage IV
TCGA-AA-3955	MALE	Not Available	638	Not Available		Colon Adenocarcinoma	Colon	Alive	T2	N2a	M0	Stage IIIB
TCGA-A6-6650	FEMALE	162.6	627	94.7	35.818862	Colon Adenocarcinoma	Colon	Alive	T3	N0	M0	Stage IIA
TCGA-AA-3712	MALE	Not Available	0	Not Available		Colon Adenocarcinoma	Colon	Alive	T3	N2	M0	Stage III
TCGA-A6-A5ZU	MALE	170	293	99.1	34.290066	Colon Adenocarcinoma	Colon	Alive	T3	N1	M0	Stage IIIB
TCGA-A6-5661	FEMALE	170	1020	69.1	23.910003	Colon Adenocarcinoma	Colon	Alive	T3	N0	M0	Stage IIA
TCGA-AA-3680	FEMALE	Not Available	335	Not Available		Colon Adenocarcinoma	Colon	Dead	T4	N2	M1	Stage IV
TCGA-F4-6854	FEMALE	170	16	66	22.837737	Colon Adenocarcinoma	Colon	Alive	T3	N0	M0	Stage IIA
TCGA-AY-A69D	FEMALE	165	543	103	37.832887	Colon Adenocarcinoma	Colon	Alive	T3	N0	M0	Stage IIA
TCGA-A6-2684	FEMALE	163	1127	70.1	26.383413	Colon Adenocarcinoma	Colon	Alive	T2	N0	M0	Stage I
TCGA-AA-3967	MALE	Not Availabl	943	Not Available		Colon Adenocarcinoma	Colon	Alive	T3	N1	M0	Stage IIIB

		e										
TCGA-AY-4071	FEMALE	Not Available	29	113.7		Not Available	Not Available	Dead	T1	N0		Stage I
TCGA-AA-3811	FEMALE	Not Available	306	Not Available		Colon Adenocarcinoma	Colon	Dead	T3	N2	M0	Stage III
TCGA-CM-5868	FEMALE	171	518	99.8	34.130016	Colon Adenocarcinoma	Colon	Alive	T4a	N1a	M1a	Stage IVA
TCGA-AA-3692	FEMALE	Not Available	1095	Not Available		Colon Mucinous Adenocarcinoma	Colon	Dead	T3	N2	M1	Stage IV
TCGA-D5-6531	MALE	167	540	60	21.513386	Colon Adenocarcinoma	Colon	Alive	T3	N0	M0	Stage IIA
TCGA-CM-6169	MALE	176	396	109.3	35.285538	Colon Adenocarcinoma	Colon	Alive	T3	N0	M0	Stage IIA
TCGA-G4-6293	FEMALE	150	4051	64.5	28.666667	Colon Adenocarcinoma	Colon	Alive	T3	N1	M0	Stage III
TCGA-CM-6163	MALE	179	427	75.8	23.657119	Colon Adenocarcinoma	Colon	Alive	T1	N0	M0	Stage I
TCGA-CK-6751	FEMALE	Not Available	518	Not Available		Colon Mucinous Adenocarcinoma	Colon	Alive	T2	N0	MX	Stage I
TCGA-CM-6677	FEMALE	167	337	99.5	35.677715	Colon Adenocarcinoma	Colon	Alive	T3	N0	M0	Stage IIA
TCGA-AZ-4616	FEMAL	Not Availabl	156	Not Available		Colon Adenocarcinoma	Colon	Dead	T3	N2	M1	Stage IV

	E	e										
TCGA-AA-3496	FEMALE	Not Available	31	Not Available		Colon Adenocarcinoma	Colon	Alive	T3	N0	M0	Stage II
TCGA-AA-3492	FEMALE	Not Available	1	Not Available		Colon Adenocarcinoma	Colon	Dead	T3	N0	M0	Stage II
TCGA-A6-6651	FEMALE	162.6	662	88.5	33.47358	Colon Adenocarcinoma	Colon	Alive	T3	N1b	MX	Stage IIIB
TCGA-AD-6548	FEMALE	157.5	650	78.6	31.68556	Colon Adenocarcinoma	Colon	Alive	T2	N0	M0	Stage I
TCGA-CM-6679	MALE	178	306	106.5	33.61318	Colon Adenocarcinoma	Colon	Alive	T3	N0	M0	Stage IIA
TCGA-AA-A02Y	MALE	Not Available	1216	Not Available		Colon Adenocarcinoma	Colon	Alive	T2	N0	M0	Stage I
TCGA-D5-6920	FEMALE	160	377	73	28.51563	Colon Adenocarcinoma	Colon	Alive	T3	N0	M0	Stage IIA
TCGA-AZ-4615	MALE	170	1002	84	29.06574	Colon Adenocarcinoma	Colon	Alive	T3	N1	M0	Stage IIIB
TCGA-DM-A1HA	MALE	163	2600	67	25.21736	Colon Adenocarcinoma	Colon	Alive	T3	N2	M0	Stage IIIC
TCGA-AA-3939	MALE	Not Available	395	Not Available		Colon Adenocarcinoma	Colon	Alive	T3	N0	M0	Stage IIA
TCGA-AA-	MAL	Not Ava	1126	Not Available		Colon Adenocarci	Colon	Alive	T2	N1	M0	Stage

3842	E	ilable				noma						IIIA
TCGA-A6-6782	MALE	175	617	63.2	20.63673	Colon Adenocarcinoma	Colon	Alive	T4a	N0	MX	Stage IIB
TCGA-AA-A02R	FEMALE	Not Available	670	Not Available		Colon Adenocarcinoma	Colon	Dead	T3	N0	M0	Stage IIA
TCGA-DM-A0XD	MALE	160	743	62	24.21875	Colon Adenocarcinoma	Colon	Dead	T3	N0	M0	Stage IIA
TCGA-A6-5667	FEMALE	175	887	76	24.81633	Colon Adenocarcinoma	Colon	Alive	T3	N1a	MX	Stage IIIB
TCGA-AA-3524	MALE	Not Available	1096	Not Available		Colon Adenocarcinoma	Colon	Alive	T3	N0	M0	Stage II
TCGA-QG-A5YX	FEMALE	163	1003	68.49	25.77816	Colon Adenocarcinoma	Colon	Alive	T3	N0	MX	Stage IIA
TCGA-AY-A71X	FEMALE	164	588	130	48.33432	Colon Adenocarcinoma	Colon	Alive	T2	N0	M0	Stage I
TCGA-AM-5821	FEMALE	148	28	68	31.04456	Colon Adenocarcinoma	Colon	Alive	T3	N0	M0	Stage IIA
TCGA-3L-AA1B	FEMALE	173	475	63.3	21.15006	Colon Adenocarcinoma	Colon	Alive	T2	N0	M0	Stage I
TCGA-AY-A54L	FEMALE	172	525	68	22.9854	Colon Adenocarcinoma	Colon	Alive	T2	N0	M0	Stage I
TCGA-AZ-	MAL	Not Ava	1776	84		Colon Adenocarci	Colon	Alive	T3	N0	M0	Stage

4315	E	ilable				noma						IIA
TCGA-AA-3525	MALE	Not Available	1	Not Available		Colon Adenocarcinoma	Colon	Alive	T3	N1	M0	Stage IIIB
TCGA-NH-A5IV	FEMALE	158.5	0	49.5	19.703655	Colon Adenocarcinoma	Colon	Alive	T3	N0	MX	Stage IIA
TCGA-A6-3810	MALE	175	1111	122.7	40.065531	Colon Adenocarcinoma	Colon	Alive	T3	N0	M0	Stage IIA
TCGA-AA-3837	MALE	Not Available	1186	Not Available		Colon Mucinous Adenocarcinoma	Colon	Alive	T3	N0	M0	Stage IIA
TCGA-AA-3815	FEMALE	Not Available	1005	Not Available		Colon Adenocarcinoma	Colon	Alive	T3	N0	M0	Stage IIA
TCGA-AA-3975	MALE	Not Available	1036	Not Available		Colon Adenocarcinoma	Colon	Alive	T2	N0	M0	Stage I
TCGA-AA-3554	FEMALE	Not Available	546	Not Available		Colon Adenocarcinoma	Colon	Alive	T3	N0	M0	Stage IIA
TCGA-AA-3660	FEMALE	Not Available	2375	Not Available		Colon Adenocarcinoma	Colon	Alive	T3	N0	M0	Stage II
TCGA-AA-3852	MALE	Not Available	0	Not Available		Colon Mucinous Adenocarcinoma	Colon	Dead	T3	N0	M0	Stage IIA
TCGA-AA-3664	FEMALE	Not Available	1643	Not Available		Colon Adenocarcinoma	Colon	Alive	T3	N0	M0	Stage II
TCGA-AA-	MAL	Not Ava	1127	Not Available		Colon Adenocarci	Colon	Alive	T3	N0	M0	Stage

3522	E	ilable				noma						IIA
TCGA-CM-6162	FEMALE	171	365	109	37.276643	Colon Mucinous Adenocarcinoma	Colon	Alive	T3	N1a	M0	Stage IIIB
TCGA-CM-6167	FEMALE	174	456	89	29.396222	Colon Adenocarcinoma	Colon	Alive	T3	N2b	M0	Stage IIIC
TCGA-G4-6317	FEMALE	162	1095	95.3	36.313306	Colon Adenocarcinoma	Colon	Alive	T3	N2	MX	Stage IIIC
TCGA-QG-A5YV	FEMALE	173	1301	109.226	36.495504	Colon Adenocarcinoma	Colon	Alive	T4b	N1a	MX	Stage IIIC
TCGA-A6-5665	FEMALE	157	671	90.8	36.837719	Colon Adenocarcinoma	Colon	Alive	T3	N0	M0	Stage IIA
TCGA-AA-3844	FEMALE	Not Available	454	Not Available		Colon Adenocarcinoma	Colon	Alive	T3	N2	M0	Stage IIIC
TCGA-CM-6161	FEMALE	174	457	65.6	21.667333	Colon Adenocarcinoma	Colon	Alive	T2	N0	M0	Stage I
TCGA-CM-5344	FEMALE	156	670	64.9	26.668331	Colon Adenocarcinoma	Colon	Alive	T3	N1b	M0	Stage IIIB
TCGA-F4-6856	MALE	160	1074	61	23.828813	Colon Mucinous Adenocarcinoma	Colon	Alive	T2	N0	M0	Stage I
TCGA-AZ-6605	MALE	Not Available	159	Not Available		Colon Adenocarcinoma	Colon	Dead	T4	N1	M0	Stage IIIB
TCGA-AA-	MAL	Not Ava	1127	Not Available		Colon Adenocarci	Colon	Alive	T3	N0	M0	Stage

3685	E	ilable				noma						II
TCGA-G4-6294	MALE	Not Available	858	47.5		Colon Adenocarcinoma	Colon	Dead	T3	N1	M1	Stage IV
TCGA-F4-6855	FEMALE	167	1442	80	28.686514	Colon Adenocarcinoma	Colon	Alive	T3	N0	M0	Stage IIA
TCGA-A6-6137	MALE	180.3	824	93	28.60826	Colon Adenocarcinoma	Colon	Alive	T3	N1c	M0	Stage IIIB
TCGA-AA-3984	FEMALE	Not Available	0	Not Available		Colon Adenocarcinoma	Colon	Alive	T3	N0	M0	Stage IIA
TCGA-AA-3818	FEMALE	Not Available	30	Not Available		Colon Adenocarcinoma	Colon	Dead	T3	N0	M0	Stage IIA
TCGA-AA-3527	FEMALE	Not Available	0	Not Available		Colon Adenocarcinoma	Colon	Alive	T3	N0	M0	Stage IIA
TCGA-AA-3989	MALE	Not Available	242	Not Available		Colon Adenocarcinoma	Colon	Dead	T3	N2	M1	Stage IV
TCGA-AZ-6601	MALE	162	3042	59	22.481133	Colon Adenocarcinoma	Colon	Dead	T3	N0	M0	Stage II
TCGA-AZ-5407	FEMALE	Not Available	2683	Not Available		Colon Adenocarcinoma	Colon	Alive	T1	N0	M0	Stage I
TCGA-AA-3870	FEMALE	Not Available	912	Not Available		Colon Adenocarcinoma	Colon	Alive	T3	N2	M1	Stage IV
TCGA-AA-	FEM	Not Ava	1158	Not Available		Colon Adenocarci	Colon	Dead	T3	N1	M0	Stage

A01P	ALE	ilable				noma						III
TCGA-DM-A28E	FEMALE	150	3648	75	33.333333	Colon Adenocarcinoma	Colon	Alive	T3	N0	M0	Stage IIA
TCGA-AZ-6598	FEMALE	Not Available	1503	Not Available		Colon Adenocarcinoma	Colon	Dead	T3	N0	MX	Stage II
TCGA-CK-5915	MALE	Not Available	0	Not Available		Colon Adenocarcinoma	Colon	Alive	T2	N0	MX	Stage I
TCGA-F4-6459	FEMALE	162	262	55	20.957177	Colon Adenocarcinoma	Colon	Dead	T3	N2a	M0	Stage IIIB
TCGA-D5-6927	MALE	177	287	76	24.258677	Colon Adenocarcinoma	Colon	Alive	T3	N0	M0	Stage IIA
TCGA-AA-3495	MALE	Not Available	1127	Not Available		Colon Adenocarcinoma	Colon	Alive	T2	N0	M0	Stage I
TCGA-AA-3511	MALE	Not Available	212	Not Available		Colon Adenocarcinoma	Colon	Alive	T4	N0	M0	Stage II
TCGA-AZ-4681	FEMALE	Not Available	3247	68		Colon Adenocarcinoma	Colon	Alive	T3	N0	M0	Stage IIA
TCGA-AA-A01T	FEMALE	Not Available	1005	Not Available		Colon Adenocarcinoma	Colon	Alive	T3	N1	M0	Stage III
TCGA-AA-3662	FEMALE	Not Available	184	Not Available		Colon Adenocarcinoma	Colon	Alive	T4	N2	M1	Stage IV
TCGA-AA-	MAL	Not Ava	549	Not Available		Colon Adenocarci	Colon	Alive	T3	N0	M0	Stage

A00K	E	ilable				noma						IIA
TCGA-A6-6780	MALE	168	612	64.1	22.71117	Colon Mucinous Adenocarcinoma	Colon	Alive	T3	N0	MX	Stage IIA
TCGA-AA-3688	MALE	Not Available	578	Not Available		Colon Adenocarcinoma	Colon	Alive	T3	N1	M1	Stage IV
TCGA-NH-A50V	MALE	167.6	588	65.9	23.46051	Colon Adenocarcinoma	Colon	Alive	T3	N2a	M0	Stage IIIB
TCGA-AA-A02K	MALE	Not Available	426	Not Available		Colon Adenocarcinoma	Colon	Dead	T4	N2	M1	Stage IV
TCGA-AA-A00A	MALE	Not Available	1157	Not Available		Colon Adenocarcinoma	Colon	Alive	T3	N0	M0	Stage IIA
TCGA-D5-6534	FEMALE	170	1316	94	32.52595	Colon Mucinous Adenocarcinoma	Colon	Alive	T3	N0	M0	Stage IIA
TCGA-D5-6539	FEMALE	165	380	63	23.1405	Colon Adenocarcinoma	Colon	Alive	T3	N0	M0	
TCGA-AA-3851	MALE	Not Available	1006	Not Available		Colon Adenocarcinoma	Colon	Alive	T3	N0	M0	Stage IIA
TCGA-AD-6965	MALE	183	805	95	28.36752	Colon Adenocarcinoma	Colon	Alive	T4a	N2b	M0	Stage IIIC
TCGA-G4-6628	MALE	169	2424	91	31.86163	Colon Adenocarcinoma	Colon	Alive	T2	N0	M0	Stage I
TCGA-AU-	FEM	157	441	83	33.67	Colon Adenocarci	Colon	Alive	T3	N0	M0	Stage

3779	ALE				277	noma						IIA
TCGA-G4-6625	FEMALE	149	2792	65.5	29.503188	Colon Adenocarcinoma	Colon	Alive	T3	N0	M0	Stage IIA
TCGA-CM-4744	MALE	183	609	89.7	26.787491	Colon Adenocarcinoma	Colon	Alive	T2	N0	M0	Stage I
TCGA-D5-6530	MALE	182	621	89	26.868874	Colon Adenocarcinoma	Colon	Alive	T2	N0	M0	Stage I
TCGA-NH-A8F7	FEMALE	80.3	543	175.3	271.86635	Colon Adenocarcinoma	Colon	Alive	T3	N0	MX	Stage IIA
TCGA-AA-3952	MALE	Not Available	61	Not Available		Colon Adenocarcinoma	Colon	Dead	T3	N2	M0	Stage IIIC
TCGA-DM-A285	FEMALE	175	179	72	23.5102	Colon Mucinous Adenocarcinoma	Colon	Dead	T3	N2	M1	Stage IV
TCGA-AU-6004	FEMALE	160	824	55	21.484438	Colon Adenocarcinoma	Colon	Alive	T2	N0	M0	Stage I
TCGA-DM-A28F	MALE	182	1094	83	25.057736	Colon Adenocarcinoma	Colon	Dead	T3	N1	M0	Stage IIIB
TCGA-CM-4752	MALE	163	396	71.2	26.798815	Colon Adenocarcinoma	Colon	Alive	T3	N0	M0	Stage IIA
TCGA-A6-5662	MALE	180	718	84.4	26.049938	Colon Adenocarcinoma	Colon	Alive	T3	N2	M1	Stage IVA
TCGA-WS-	FEM	152	2130	34	14.71	Colon Mucinous	Colon	Alive	T3	N0	MX	Stage

AB45	ALE				607	Adenocarcinoma						IIA
TCGA-RU-A8FL	MALE	187.96	1177	125	35.38174	Colon Adenocarcinoma	Colon	Alive	T3	N2a	MX	Stage IIIB
TCGA-AA-A02W	FEMALE	Not Available	1247	Not Available		Colon Adenocarcinoma	Colon	Alive	T2	N0	M0	Stage I
TCGA-CA-5254	FEMALE	154	386	55	23.19109	Colon Adenocarcinoma	Colon	Alive	T3	N0	M0	Stage IIA
TCGA-D5-6930	MALE	184	406	90	26.58318	Colon Mucinous Adenocarcinoma	Colon	Alive	T3	N0	M0	Stage IIA
TCGA-DM-A28K	MALE	174	2988	78	25.76298	Colon Mucinous Adenocarcinoma	Colon	Alive	T3	N0	M0	Stage IIA
TCGA-AA-3860	FEMALE	Not Available	945	Not Available		Colon Adenocarcinoma	Colon	Alive	T3	N1	M0	Stage IIIB
TCGA-D5-6932	MALE	168	346	75	26.57313	Colon Adenocarcinoma	Colon	Alive	T3	N0	M0	Stage IIA
TCGA-AA-3819	FEMALE	Not Available	761	Not Available		Colon Adenocarcinoma	Colon	Alive	T3	N0	M0	Stage IIA
TCGA-AA-3872	MALE	Not Available	0	Not Available		Colon Adenocarcinoma	Colon	Alive	T4	N2	M1	Stage IV
TCGA-A6-A56B	MALE	180	1711	77.2	23.82716	Colon Adenocarcinoma	Colon	Dead	T3	N1	M0	Stage IIIB
TCGA-AA-	FEM	Not Ava	61	Not Available		Colon Adenocarci	Colon	Dead	T3	N2	M1	Stage

A02H	ALE	ilable				noma						IV
TCGA-AA-3532	MALE	Not Available	882	Not Available		Colon Adenocarcinoma	Colon	Alive	T3	N0	M0	Stage IIA
TCGA-D5-6931	MALE	178	365	70	22.093317	Colon Adenocarcinoma	Colon	Alive	T4b	N2	M0	Stage IIIC
TCGA-AZ-4308	FEMALE	Not Available	3324	73		Colon Adenocarcinoma	Colon	Alive	T3	N1	M0	Stage IIIB
TCGA-AZ-6603	FEMALE	Not Available	899	Not Available		Colon Adenocarcinoma	Colon	Dead	T2	N1	MX	

Table S3. The detailed clinical information for dataset TCGA-READ.

A0_Samples	A18_Sex	A19_Height	A1_OS	A20_Weight	A21_BMI	A22_histological_type	A23_Tumor_tissue_site	A2_Event	A3_T	A4_N	A5_M	A6_Stage
TCGA-AG-A002	MALE	Not Available	638	Not Available		Rectal Adenocarcinoma	Rectum	Alive	T2	N0	M0	Stage I
TCGA-AG-3890	MALE	Not Available	518	Not Available		Rectal Adenocarcinoma	Rectum	Alive	T2	N0	M0	Stage I
TCGA-EI-6511	MALE	165	482	69	25.343435	Rectal Adenocarcinoma	Rectum	Alive	T3	N1	M0	Stage IIIB
TCGA-AG-A01J	FEMALE	Not Available	31	Not Available		Rectal Adenocarcinoma	Rectum	Alive	T3	N0	M0	Stage IIA
TCGA-	MALE	Not Ava	485	Not Available		Rectal Adenocarci	Rectum	Alive	T2	N0	M0	Stage

AG-A014		ilable				noma						I
TCGA-DC-4745	FEMALE	165	639	93.8	34.453363	Rectal Adenocarcinoma	Rectum	Alive	T3	N1a	M0	Stage IIIB
TCGA-AG-A015	FEMALE	Not Available	1096	Not Available		Rectal Adenocarcinoma	Rectum	Alive	T1	N0	M0	Stage I
TCGA-AF-2691	FEMALE	167.6	1309	52.4	18.654448	Rectal Adenocarcinoma	Rectum	Alive	T1	N0	M0	Stage I
TCGA-AG-3891	FEMALE	Not Available	548	Not Available		Rectal Adenocarcinoma	Rectum	Alive	T2	N0	M0	Stage I
TCGA-DC-5337	MALE	165	792	69.4	25.491288	Rectal Adenocarcinoma	Rectum	Alive	T1	N0	M0	Stage I
TCGA-AG-4015	FEMALE	Not Available	0	Not Available		Rectal Adenocarcinoma	Rectum	Alive	T3	N0	M0	Stage IIA
TCGA-DC-6158	MALE	160	334	71.7	28.007781	Rectal Adenocarcinoma	Rectum	Dead	T2	N0	M0	Stage I
TCGA-DY-A1DE	FEMALE	160	3932	70	27.343375	Rectal Adenocarcinoma	Rectum	Alive	T3	N0	M0	Stage IIA
TCGA-AF-3912	Not Available	Not Available		Not Available		Not Available	Not Available					
TCGA-AG-A02G	MALE	Not Available	1185	Not Available		Rectal Adenocarcinoma	Rectum	Dead	T2	N1	M1	Stage IV
TCGA-	MALE	180.3	1155	107.6	33.09	Not Available	Not Availabl	Alive	T2	N0	MX	Stage

AF-2693					945		e					I
TCGA-AG-4009	MALE	Not Available	426	Not Available		Rectal Adenocarcinoma	Rectum	Alive	T2	N0	M0	Stage I
TCGA-EI-6513	MALE	175	497	78	25.469399	Rectal Adenocarcinoma	Rectum	Alive	T3	N1	M0	Stage IIIB
TCGA-AG-A00H	MALE	Not Available	790	Not Available		Rectal Adenocarcinoma	Rectum	Alive	T3	N0	M0	Stage IIA
TCGA-DT-5265	MALE	Not Available	384	Not Available		Rectal Mucinous Adenocarcinoma	Rectum	Alive	T3	N0	M0	Stage II
TCGA-AG-3586	FEMALE	Not Available	31	Not Available		Not Available	Rectum	Alive	T3	N2	M0	Stage III
TCGA-BM-6198	MALE	Not Available	646	119		Rectal Adenocarcinoma	Rectum	Alive	T3	N1		Stage IIIB
TCGA-AF-A56K	MALE	182.5	2635	145.5	43.68549	Rectal Adenocarcinoma	Rectum	Alive	T3	N0	M0	Stage IIA
TCGA-DC-6157	MALE	185	1581	116	33.89335	Rectal Adenocarcinoma	Rectum	Alive	T2	N0	M0	Stage I
TCGA-AG-3893	MALE	Not Available	1065	Not Available		Rectal Adenocarcinoma	Rectum	Alive	T3	N1	M0	Stage IIIB
TCGA-AF-3914	MALE	172.7	1146	64.2	21.525536	Rectal Adenocarcinoma	Rectum	Alive	T3	N2a	M0	Stage IIIB
TCGA-EI-	FEMA	162	625	65	24.76	Rectal Adenocarci	Rectum	Alive	T3	N0	M0	Stage

6506	LE				757	noma						IIA
TCGA-EI-6508	FEMALE	159	636	66	26.106566	Rectal Adenocarcinoma	Rectum	Alive	T3	N1a	M0	Stage IIIB
TCGA-AH-6644	MALE	Not Available	838	Not Available		Rectal Adenocarcinoma	Rectum	Alive	T3	NX	MX	
TCGA-EI-6884	MALE	166	328	73	26.491511	Rectal Adenocarcinoma	Rectum	Alive	T3	N1	M0	Stage IIIA
TCGA-CL-5917	FEMALE	Not Available	2376	Not Available		Rectal Adenocarcinoma	Rectum	Alive	T3	N2	MX	Stage IIIC
TCGA-EI-7002	MALE	185	364	93	27.173122	Rectal Adenocarcinoma	Rectum	Alive	T3	N2	M1	Stage IV
TCGA-CI-6623	MALE	Not Available	1443	Not Available		Rectal Adenocarcinoma	Rectum	Alive	T1	N0	M0	Stage I
TCGA-CL-4957	FEMALE	Not Available	425	Not Available		Rectal Adenocarcinoma	Rectum	Alive	T3	N1	M0	
TCGA-G5-6233	MALE	176.9	556	97.7	31.220443	Rectal Adenocarcinoma	Rectum	Dead	T3	N2	M0	
TCGA-F5-6812	MALE	188	1110	90	25.464401	Rectal Adenocarcinoma	Rectum	Alive	T3	N0	M0	Stage IIA
TCGA-AG-3574	FEMALE	Not Available	91	Not Available		Rectal Adenocarcinoma	Rectum	Dead	T3	N0	M0	Stage II
TCGA-	FEMA	Not Ava	943	Not Available		Rectal Adenocarci	Rectum	Alive	T2	N0	M1	Stage

AG-A01N	LE	ilable				noma						IV
TCGA-AG-3612	FEMALE	Not Available	608	Not Available		Rectal Adenocarcinoma	Rectum	Alive	T3	N1	M0	Stage IIIB
TCGA-AG-A023	FEMALE	Not Available	1581	Not Available		Rectal Adenocarcinoma	Rectum	Dead	T4	N2	M1	Stage IV
TCGA-AG-A01L	MALE	Not Available	0	Not Available		Rectal Adenocarcinoma	Rectum	Alive	T3	N1	M0	Stage IIIB
TCGA-F5-6861	FEMALE	162	1160	64	24.386653	Rectal Adenocarcinoma	Rectum	Alive	T3	N0	M0	Stage IIA
TCGA-EI-6917	MALE	176	531	80	25.826645	Rectal Adenocarcinoma	Rectum	Alive	T3	N1	M0	Stage IIIA
TCGA-AG-3598	MALE	Not Available	1522	Not Available		Rectal Adenocarcinoma	Rectum	Alive	T3	N0	M0	Stage IIA
TCGA-AG-3883	MALE	Not Available	31	Not Available		Rectal Adenocarcinoma	Rectum	Alive	T2	N0	M0	Stage I
TCGA-CI-6619	MALE	Not Available	184	Not Available		Rectal Adenocarcinoma	Rectum	Alive	T3	N1c	M1	Stage IV
TCGA-AH-6897	MALE	180.5	804	86.4	26.519913	Rectal Adenocarcinoma	Rectum	Alive	T2	N0	M0	Stage I
TCGA-AG-4007	MALE	Not Available	31	Not Available		Rectal Adenocarcinoma	Rectum	Alive	T4	N2	M1	Stage IV
TCG	FE	Not	15	Not		Rectal	Rectum	Ali	T1	N0	M0	Sta

A-AG-A025	MALE	Available	20	Available		Adenocarcinoma		ve				ge I
TCGA-DY-A1DG	MALE	175	1566	86	28.081163	Rectal Adenocarcinoma	Rectum	Dead	T3	N1	M1	Stage IVA
TCGA-DC-6682	MALE	184	762	172.4	50.921155	Rectal Adenocarcinoma	Rectum	Alive	T3	N0	M0	Stage IIA
TCGA-DC-6683	MALE	183	762	82.6	24.664482	Rectal Adenocarcinoma	Rectum	Alive	T3	N1b	M0	Stage IIIB
TCGA-AG-3901	FEMALE	Not Available	761	Not Available		Rectal Mucinous Adenocarcinoma	Rectum	Alive	T3	N1	M0	Stage IIIB
TCGA-AG-A020	FEMALE	Not Available	31	Not Available		Rectal Mucinous Adenocarcinoma	Rectum	Alive	T3	N1	M0	Stage III
TCGA-AF-A56N	FEMALE	165.1	360	78.1	28.652133	Rectal Adenocarcinoma	Rectum	Alive	T3	N0	M0	Stage IIA
TCGA-DC-6160	MALE	175.4	1339	100.1	32.5368	Rectal Adenocarcinoma	Rectum	Alive	T2	N0	M0	Stage I
TCGA-AF-6655	MALE	173	609	53.2	17.7754	Rectal Adenocarcinoma	Rectum	Alive	T2	N1	M0	Stage IIIA
TCGA-AG-3575	MALE	Not Available	365	Not Available		Rectal Adenocarcinoma	Rectum	Alive	T3	N0	M0	Stage II
TCGA-EI-6883	MALE	180	350	73	22.5308	Rectal Adenocarcinoma	Rectum	Alive	T3	N0	M0	Stage IIC

					6							
TCGA-AF-3400	MALE	177.8	1049	85.4	27.014344	Rectal Mucinous Adenocarcinoma	Rectum	Alive	T3	N0	M0	Stage IIA
TCGA-AG-3583	MALE	Not Available	610	Not Available		Rectal Mucinous Adenocarcinoma	Rectum	Dead	T3	N2	M1	Stage IV
TCGA-AG-3584	MALE	Not Available	730	Not Available		Rectal Adenocarcinoma	Rectum	Dead	T3	N2	M1	Stage IV
TCGA-CI-6620	FEMALE	Not Available	1009	Not Available		Rectal Adenocarcinoma	Rectum	Alive	T3	N1	M1	Stage IVA
TCGA-DC-5869	FEMALE	170	943	62.4	21.5917	Rectal Adenocarcinoma	Rectum	Alive	T3	N1a	M0	Stage IIIB
TCGA-G5-6235	MALE	169	1696	77.6	27.16992	Rectal Adenocarcinoma	Rectum	Alive	T3	N1	MX	Stage IIIB
TCGA-AF-4110	MALE	188	912	92	26.029888	Not Available	Not Available	Alive	T4a	N2b	MX	Stage IVA
TCGA-AG-3581	MALE	Not Available	215	Not Available		Rectal Adenocarcinoma	Rectum	Alive	T2	N0	M0	Stage I
TCGA-AF-3913	MALE	180	316	95.5	29.475331	Rectal Adenocarcinoma	Rectum	Dead	T3	N1	M1	Stage IV
TCGA-AF-2692	FEMALE	170	412	64.4	22.283374	Not Available	Not Available	Alive	T3	N0	M0	Stage IIA
TCGA-AG-	FEMALE	Not Availabl	1035	Not Available		Rectal Adenocarcinoma	Rectum	Alive	T3	N0	M0	Stage IIA

3593		e										
TCGA-AG-3591	FEMALE	Not Available	1035	Not Available		Rectal Adenocarcinoma	Rectum	Alive	T3	N0	M0	Stage IIA
TCGA-F5-6702	MALE	172	869	76	25.68956	Rectal Adenocarcinoma	Rectum	Dead	T3	N1	M1	Stage IVA
TCGA-AG-3898	MALE	Not Available	1461	Not Available		Rectal Adenocarcinoma	Rectum	Alive	T3	N0	M0	Stage IIA
TCGA-AG-A036	MALE	Not Available	3562	Not Available		Rectal Adenocarcinoma	Rectum	Alive	T3	N2	M0	Stage III
TCGA-AG-3599	MALE	Not Available	366	Not Available		Not Available	Rectum	Alive	T2	N0	M0	Stage I
TCGA-AG-4005	MALE	Not Available	427	Not Available		Rectal Adenocarcinoma	Rectum	Alive	T3	N2	M1	Stage IV
TCGA-AG-3902	MALE	Not Available	974	Not Available		Rectal Adenocarcinoma	Rectum	Alive	T3	N0	M0	Stage IIA
TCGA-AG-3906	FEMALE	Not Available	608	Not Available		Rectal Adenocarcinoma	Rectum	Alive	T2	N0	M0	Stage I
TCGA-AG-4001	FEMALE	Not Available	1096	Not Available		Rectal Adenocarcinoma	Rectum	Alive	T3	N0	M0	Stage IIA
TCGA-AG-3580	MALE	Not Available	244	Not Available		Rectal Adenocarcinoma	Rectum	Alive	T1	N0	M0	Stage I
TCGA-AF-	FEMALE	165	512	96.6	35.4820	Rectal Adenocarcinoma	Rectum	Dead	T2	N0	M0	Stage I

5654					9							
TCGA-AG-3601	MALE	Not Available	0	Not Available		Rectal Adenocarcinoma	Rectum	Alive	T4	N1	M0	Stage IIIB
TCGA-AG-3999	FEMALE	Not Available	853	Not Available		Rectal Adenocarcinoma	Rectum	Alive	T3	N2	M0	Stage IIIC
TCGA-AF-3911	MALE	180.3	1148	94.5	29.069969	Rectal Adenocarcinoma	Rectum	Alive	T3	N2	MX	Stage IIIC
TCGA-DY-A1DD	FEMALE	156	1741	42	17.258388	Rectal Adenocarcinoma	Rectum	Dead	T3	N1	M0	Stage IIIB
TCGA-G5-6641	MALE	189.3	804	118.6	33.096661	Rectal Mucinous Adenocarcinoma	Rectum	Alive	T1	N1	MX	Stage IIIA
TCGA-AG-3725	FEMALE	Not Available	0	Not Available		Rectal Adenocarcinoma	Rectum	Alive	T3	N2	M0	Stage III
TCGA-AG-4008	MALE	Not Available	518	Not Available		Rectal Adenocarcinoma	Rectum	Alive	T3	N0	M0	Stage IIA
TCGA-AG-3882	FEMALE	Not Available	608	Not Available		Rectal Adenocarcinoma	Rectum	Alive	T2	N0	M0	Stage I
TCGA-EI-6512	FEMALE	170	538	61	21.107727	Rectal Adenocarcinoma	Rectum	Alive	T3	N1	M0	Stage IIIB
TCGA-EI-6882	MALE	178	262	108	34.086661	Rectal Mucinous Adenocarcinoma	Rectum	Alive	T3	N0	M0	Stage IIA
TCGA-	FEMA	Not Ava	0	Not Available		Rectal Adenocarci	Rectum	Alive	T3	N0	M0	Stage

AG-A01W	LE	ilable				noma						II
TCGA-F5-6464	FEMALE	159	303	68	26.897767	Rectal Adenocarcinoma	Rectum	Dead	T4b	N2a	M0	Stage IIIC
TCGA-AG-3727	FEMALE	Not Available	30	Not Available		Rectal Adenocarcinoma	Rectum	Alive	T3	N1	M0	Stage III
TCGA-AG-3909	FEMALE	Not Available	608	Not Available		Rectal Adenocarcinoma	Rectum	Alive	T3	N1	M0	Stage IIIB
TCGA-F5-6864	FEMALE	157	379	78	31.644429	Rectal Adenocarcinoma	Rectum	Alive	T3	N2a	M0	Stage IIIB
TCGA-AG-3582	MALE	Not Available	1096	Not Available		Rectal Adenocarcinoma	Rectum	Dead	T3	N1	M1	Stage IV
TCGA-AG-3594	MALE	Not Available	61	Not Available		Rectal Mucinous Adenocarcinoma	Rectum	Dead	T3	N0	M0	Stage IIA
TCGA-AF-6136	FEMALE	161.3	741	67.2	25.828558	Rectal Adenocarcinoma	Rectum	Alive	T3	N1b	MX	Stage IIIB
TCGA-AG-3878	MALE	Not Available	30	Not Available		Rectal Adenocarcinoma	Rectum	Alive	T2	N0	M0	Stage I
TCGA-G5-6572	MALE	185.4	1432	140	40.729945	Rectal Adenocarcinoma	Rectum	Dead				
TCGA-AG-A00C	FEMALE	Not Available	183	Not Available		Rectal Adenocarcinoma	Rectum	Alive	T3	N1	M0	Stage IIIB
TCG	FE	Not	31	Not		Rectal	Rectum	Ali	T2	N0	M0	Sta

A-AG-3896	MALE	Available		Available		Adenocarcinoma		ve				ge I
TCGA-EI-6514	FEMALE	Not Available	496	Not Available		Rectal Adenocarcinoma	Rectum	Alive	T3	N0	M0	Stage IIA
TCGA-AH-6547	FEMALE	Not Available	76	Not Available		Rectal Adenocarcinoma	Rectum	Dead	T3	N0	MX	
TCGA-AH-6549	MALE	Not Available	532	Not Available		Rectal Adenocarcinoma	Rectum	Alive	T3	NX	MX	
TCGA-AG-3609	FEMALE	Not Available	608	Not Available		Rectal Adenocarcinoma	Rectum	Alive	T3	N2	M0	Stage IIIC
TCGA-F5-6813	MALE	174	598	85	28.07504	Rectal Adenocarcinoma	Rectum	Dead	T4a	N2	M0	Stage IIIC
TCGA-AG-A00Y	MALE	Not Available	700	Not Available		Rectal Adenocarcinoma	Rectum	Alive	T3	N0	M0	Stage IIA
TCGA-EF-5831	MALE	162	127	60	22.86237	Rectal Adenocarcinoma	Rectum	Alive	T3	N0	M0	Stage IIA
TCGA-AF-2687	MALE	163	1427	68.2	25.66901	Rectal Adenocarcinoma	Rectum	Alive	T3	N2	M0	Stage IIIC
TCGA-AH-6544	MALE	177.8	1173	91	28.78577	Rectal Adenocarcinoma	Rectum	Alive	T3	N1	M1	
TCGA-AG-A008	FEMALE	Not Available	424	Not Available		Rectal Mucinous Adenocarcinoma	Rectum	Alive	T2	N0	M0	Stage I
TCG	FE	165	97	88	32.	Rectal	Rectum	Ali	T3	N0	M0	Sta

A-F5-6811	MALE		9		32323	Adenocarcinoma		ve				ge IIA
TCGA-DC-6156	MALE	175	943	86.1	28.114429	Rectal Adenocarcinoma	Rectum	Alive	T4a	N2b	M1a	Stage IVA
TCGA-EI-7004	FEMALE	172	257	88	29.745581	Rectal Mucinous Adenocarcinoma	Rectum	Alive	T4a	N2b	M0	
TCGA-DC-6681	FEMALE	151	790	63.1	27.67422	Rectal Adenocarcinoma	Rectum	Alive	T3	N2a	M1a	Stage IVA
TCGA-AG-3578	FEMALE	Not Available	974	Not Available		Rectal Mucinous Adenocarcinoma	Rectum	Alive	T3	N0	M0	Stage IIA
TCGA-AG-3600	MALE	Not Available	184	Not Available		Rectal Adenocarcinoma	Rectum	Alive	T3	N2	M0	Stage IIIC
TCGA-AG-3892	FEMALE	Not Available	396	Not Available		Rectal Adenocarcinoma	Rectum	Alive	T1	N0	M0	Stage I
TCGA-AG-4021	FEMALE	Not Available	121	Not Available		Rectal Adenocarcinoma	Rectum	Dead	T3	N2	M1	Stage IV
TCGA-EI-6881	MALE	176	499	86	27.763343	Rectal Adenocarcinoma	Rectum	Alive	T3	N1	M0	Stage IIIA
TCGA-CI-6622	MALE	Not Available	1362	Not Available		Rectal Adenocarcinoma	Rectum	Alive	T4	N0	M0	Stage IIB
TCGA-DY-A1DC	FEMALE	Not Available	1258	68		Rectal Adenocarcinoma	Rectum	Dead	T3	N0	M0	Stage IIA

TCGA-CL-5918	FEMALE	Not Available	0	Not Available		Rectal Adenocarcinoma	Rectum	Alive	T3	N0	MX	Stage IIA
TCGA-EI-6509	MALE	178	517	82	25.880557	Rectal Adenocarcinoma	Rectum	Alive	T3	N2b	M0	Stage IIIC
TCGA-F5-6863	FEMALE	166	361	77	27.9431	Rectal Adenocarcinoma	Rectum	Dead	T4a	N1	M0	Stage IIIB
TCGA-AG-A016	MALE	Not Available	276	Not Available		Rectal Adenocarcinoma	Rectum	Alive	T3	N2	M1	Stage IV
TCGA-AG-3887	MALE	Not Available	1124	Not Available		Rectal Mucinous Adenocarcinoma	Rectum	Alive	T3	N0	M0	Stage IIA
TCGA-AG-3592	MALE	Not Available	1035	Not Available		Rectal Adenocarcinoma	Rectum	Alive	T3	N0	M0	Stage IIA
TCGA-AG-3602	FEMALE	Not Available	0	Not Available		Rectal Adenocarcinoma	Rectum	Alive	T3	N2	M0	Stage IIIC
TCGA-EI-6507	MALE	176	607	54	17.43285	Rectal Adenocarcinoma	Rectum	Alive	T3	N0	M0	Stage IIA
TCGA-AG-3894	MALE	Not Available	426	Not Available		Rectal Adenocarcinoma	Rectum	Alive	T3	N0	M0	Stage IIA
TCGA-AG-3611	MALE	Not Available	424	Not Available		Rectal Adenocarcinoma	Rectum	Alive	T3	N0	M0	Stage IIA
TCGA-DY-A0XA	FEMALE	159	3846	64	25.31545	Rectal Adenocarcinoma	Rectum	Alive	T3	N0	M0	Stage IIA

TCGA-F5-6571	FEMALE	166	1288	80	29.03179	Rectal Adenocarcinoma	Rectum	Alive	T3	N0	M0	Stage IIA
TCGA-AG-3742	FEMALE	Not Available	30	Not Available		Rectal Adenocarcinoma	Rectum	Alive	T1	N0	M0	Stage I
TCGA-AF-6672	MALE	172.7	748	93.8	31.44982	Rectal Adenocarcinoma	Rectum	Alive	T4a	N2b	MX	Stage IV
TCGA-AF-A56L	FEMALE	159	2007	74.3	29.38966	Rectal Adenocarcinoma	Rectum	Alive	T3	N2	M0	Stage IIIC
TCGA-AG-3731	MALE	Not Available	1126	Not Available		Rectal Adenocarcinoma	Rectum	Alive	T3	N1	M1	Stage IV
TCGA-DC-6155	FEMALE	163	425	55.3	20.81373	Rectal Adenocarcinoma	Rectum	Alive	T2	N1b	M0	Stage IIIA
TCGA-EF-5830	MALE	182	106	93	28.07632	Rectal Adenocarcinoma	Rectum	Alive	T4a	N0	M0	Stage IIB
TCGA-AF-2689	FEMALE	163	1201	55.5	20.88901	Rectal Adenocarcinoma	Rectum	Dead	T3	N2	M1	Stage IV
TCGA-AG-A02N	MALE	Not Available	1885	Not Available		Rectal Adenocarcinoma	Rectum	Alive	T3	N0	M0	Stage II
TCGA-DY-A1H8	FEMALE	160	992	40	15.625	Rectal Adenocarcinoma	Rectum	Dead	T2	N1	M0	Stage IIIA
TCGA-AG-	FEMALE	Not Availabl	546	Not Available		Rectal Adenocarcinoma	Rectum	Alive	T3	N1	M0	Stage IIIB

3885		e										
TCGA-AG-A01Y	FEMALE	Not Available	0	Not Available		Rectal Adenocarcinoma	Rectum	Alive	T3	N0	M0	Stage II
TCGA-AG-3728	MALE	Not Available	912	Not Available		Rectal Adenocarcinoma	Rectum	Alive	T3	N1	M0	Stage IIIB
TCGA-AH-6643	MALE	180.5	1314	84.4	25.90526	Rectal Adenocarcinoma	Rectum	Dead	T3	N2b	M0	Stage IIIC
TCGA-DY-A1DF	FEMALE	156	734	75	30.818554	Rectal Adenocarcinoma	Rectum	Dead	T3	N2	M0	Stage IIIC
TCGA-AG-3881	FEMALE	Not Available	579	Not Available		Not Available	Rectum	Alive	T3	N0	M0	Stage IIA
TCGA-AF-2690	FEMALE	160	524	54.5	21.289062	Rectal Adenocarcinoma	Rectum	Dead	T3	N2	M0	Stage IIIC
TCGA-AG-3587	MALE	Not Available	1400	Not Available		Rectal Adenocarcinoma	Rectum	Alive	T2	N0	M0	Stage I
TCGA-AG-3732	FEMALE	Not Available	1003	Not Available		Rectal Adenocarcinoma	Rectum	Alive	T2	N0	M0	Stage I
TCGA-CI-6621	MALE	Not Available	419	Not Available		Rectal Adenocarcinoma	Rectum	Alive	T3	N1	MX	Stage IIIB
TCGA-F5-6814	MALE	175	1131	61	19.918837	Rectal Adenocarcinoma	Rectum	Alive	T3	N0	M0	Stage IIA
TCGA-CI-	FEMALE	Not Availabl	1466	Not Available		Rectal Adenocarcinoma	Rectum	Alive	T2	N0	M0	Stage I

6624		e										
TCGA-AG-A02X	MALE	Not Available	1247	Not Available		Rectal Adenocarcinoma	Rectum	Alive	T2	N0	M0	Stage I
TCGA-AG-3605	FEMALE	Not Available	30	Not Available		Rectal Adenocarcinoma	Rectum	Alive	T3	N1	M1	Stage IV
TCGA-F5-6465	FEMALE	170	1506	62	21.453329	Rectal Adenocarcinoma	Rectum	Alive	T3	N0	M0	Stage IIA
TCGA-EI-6510	FEMALE	150	556	59	26.222222	Rectal Adenocarcinoma	Rectum	Alive	T2	N0	MX	
TCGA-AG-3726	FEMALE	Not Available	243	Not Available		Rectal Adenocarcinoma	Rectum	Alive	T2	N0	M0	Stage I
TCGA-EI-6885	FEMALE	164	415	53	19.705553	Rectal Adenocarcinoma	Rectum	Alive	T3	N1b	M1	Stage IV
TCGA-AG-4022	FEMALE	Not Available	1400	Not Available		Rectal Adenocarcinoma	Rectum	Alive	T3	N0	M0	Stage II
TCGA-DC-6154	FEMALE	162	365	59.4	22.633374	Rectal Adenocarcinoma	Rectum	Alive	T4a	N2b	M1	Stage IVA
TCGA-AG-A026	MALE	Not Available	59	Not Available		Rectal Adenocarcinoma	Rectum	Dead	T4	N0	M0	Stage II
TCGA-AG-3608	FEMALE	Not Available	485	Not Available		Rectal Adenocarcinoma	Rectum	Alive	T3	N0	M0	Stage IIA
TCGA-AG-	MALE	Not Availabl	1157	Not Available		Rectal Adenocarcinoma	Rectum	Alive	T3	N1	M0	Stage IIIB

A032		e										
TCGA-AG-A011	MALE	Not Available	1126	Not Available		Rectal Adenocarcinoma	Rectum	Alive	T3	N0	M0	Stage IIA
TCGA-AH-6903	MALE	183	592	74.8	22.33569	Rectal Mucinous Adenocarcinoma	Rectum	Alive	T3	N1b	M0	Stage IIIB
TCGA-DC-4749	MALE	179	762	87.1	27.18392	Rectal Adenocarcinoma	Rectum	Alive	T2	N0	M0	Stage I

Table S4. The detailed clinical information for dataset GSE147597.

title	geo_accession	organism_ch1	age:ch1	disease state:ch1	gender
Patient1 without LM primary CRC cancer tissue (circRNA)	GSM4435040	Homo sapiens	age: 65	colorectal cancer (CRC) without liver metastasis	male
Patient2 without LM primary CRC cancer tissue (circRNA)	GSM4435041	Homo sapiens	age: 72	colorectal cancer (CRC) without liver metastasis	male
Patient3 without LM primary CRC cancer tissue (circRNA)	GSM4435042	Homo sapiens	age: 67	colorectal cancer (CRC) without liver metastasis	female
Patient4 without LM primary CRC cancer tissue (circRNA)	GSM4435043	Homo sapiens	age: 61	colorectal cancer (CRC) without liver metastasis	female
Patient5 without LM primary CRC cancer tissue (circRNA)	GSM4435044	Homo sapiens	age: 59	colorectal cancer (CRC) without liver metastasis	male
Patient6 without LM primary CRC cancer tissue (circRNA)	GSM4435045	Homo sapiens	age: 74	colorectal cancer (CRC) without liver metastasis	male
Patient7 without LM primary CRC cancer tissue (circRNA)	GSM4435046	Homo sapiens	age: 69	colorectal cancer (CRC) without liver metastasis	female
Patient8 without LM primary CRC cancer tissue (circRNA)	GSM4435047	Homo sapiens	age: 66	colorectal cancer (CRC) without liver metastasis	male
Patient9 without LM	GSM44	Homo	age:	colorectal cancer (CRC)	mal

primary CRC cancer tissue (circRNA)	35048	sapiens	56	without liver metastasis	e
Patient10 without LM primary CRC cancer tissue (circRNA)	GSM4435049	Homo sapiens	age: 60	colorectal cancer (CRC) without liver metastasis	female
Patient1 with LM primary CRC cancer tissue (circRNA)	GSM4435050	Homo sapiens	age: 66	colorectal cancer (CRC) with liver metastasis	male
Patient2 with LM primary CRC cancer tissue (circRNA)	GSM4435051	Homo sapiens	age: 52	colorectal cancer (CRC) with liver metastasis	male
Patient3 with LM primary CRC cancer tissue (circRNA)	GSM4435052	Homo sapiens	age: 80	colorectal cancer (CRC) with liver metastasis	male
Patient4 with LM primary CRC cancer tissue (circRNA)	GSM4435053	Homo sapiens	age: 70	colorectal cancer (CRC) with liver metastasis	female
Patient5 with LM primary CRC cancer tissue (circRNA)	GSM4435054	Homo sapiens	age: 57	colorectal cancer (CRC) with liver metastasis	male
Patient6 with LM primary CRC cancer tissue (circRNA)	GSM4435055	Homo sapiens	age: 43	colorectal cancer (CRC) with liver metastasis	female
Patient7 with LM primary CRC cancer tissue (circRNA)	GSM4435056	Homo sapiens	age: 67	colorectal cancer (CRC) with liver metastasis	male
Patient8 with LM primary CRC cancer tissue (circRNA)	GSM4435057	Homo sapiens	age: 67	colorectal cancer (CRC) with liver metastasis	female
Patient9 with LM primary CRC cancer tissue (circRNA)	GSM4435058	Homo sapiens	age: 83	colorectal cancer (CRC) with liver metastasis	female
Patient10 with LM primary CRC cancer tissue (circRNA)	GSM4435059	Homo sapiens	age: 34	colorectal cancer (CRC) with liver metastasis	female

Table S5. The detailed clinical information for dataset GSE50117.

title	geo_accession	characteristics_ch1	characteristics_ch1.2	description	gender:ch1	localisation:ch1	tissue:ch1
CRC vs NOR	GSM1214408	tissue: adenocar	localisation: left	colorectal	male	left colon	adenocarcinoma

colorectal adenocarcinoma, patient 108		cinoma	colon	adenocarcinoma biopsy			
CRC vs NOR colorectal adenocarcinoma, patient 115	GSM1214409	tissue: adenocarcinoma	localisation: sigmoid colon	colorectal adenocarcinoma biopsy	male	sigmoid colon	adenocarcinoma
CRC vs NOR colorectal adenocarcinoma, patient 117	GSM1214410	tissue: adenocarcinoma	localisation: right colon	colorectal adenocarcinoma biopsy	male	right colon	adenocarcinoma
CRC vs NOR colorectal adenocarcinoma, patient 118	GSM1214411	tissue: adenocarcinoma	localisation: right angle	colorectal adenocarcinoma biopsy	male	right angle	adenocarcinoma
CRC vs NOR colorectal adenocarcinoma, patient 120	GSM1214412	tissue: adenocarcinoma	localisation: right colon	colorectal adenocarcinoma biopsy	female	right colon	adenocarcinoma
CRC vs NOR colorectal adenocarcinoma, patient 123	GSM1214413	tissue: adenocarcinoma	localisation: cecum	colorectal adenocarcinoma biopsy	male	cecum	adenocarcinoma
CRC vs NOR colorectal adenocarcinoma, patient 132	GSM1214414	tissue: adenocarcinoma	localisation: cecum	colorectal adenocarcinoma biopsy	female	cecum	adenocarcinoma
CRC vs NOR colorectal adenocarci	GSM1214415	tissue: adenocarcinoma	localisation: right colon	colorectal adenocarcinoma	female	right colon	adenocarcinoma

noma, patient 86				biopsy			
CRC vs NOR colorectal adenocarcinoma, patient 89	GSM1214416	tissue: adenocarcinoma	localisation: rectum / anal canal	colorectal adenocarcinoma biopsy	female	rectum / anal canal	adenocarcinoma
CRC vs NOR colorectal normal mucosa, patient 108	GSM1214417	tissue: normal mucosa	localisation: left colon	colorectal normal mucosa biopsy	male	left colon	normal mucosa
CRC vs NOR colorectal normal mucosa, patient 115	GSM1214418	tissue: normal mucosa	localisation: sigmoid colon	colorectal normal mucosa biopsy	male	sigmoid colon	normal mucosa
CRC vs NOR colorectal normal mucosa, patient 117	GSM1214419	tissue: normal mucosa	localisation: right colon	colorectal normal mucosa biopsy	male	right colon	normal mucosa
CRC vs NOR colorectal normal mucosa, patient 118	GSM1214420	tissue: normal mucosa	localisation: right angle	colorectal normal mucosa biopsy	male	right angle	normal mucosa
CRC vs NOR colorectal normal mucosa, patient 120	GSM1214421	tissue: normal mucosa	localisation: right colon	colorectal normal mucosa biopsy	female	right colon	normal mucosa
CRC vs NOR colorectal normal mucosa, patient 123	GSM1214422	tissue: normal mucosa	localisation: cecum	colorectal normal mucosa biopsy	male	cecum	normal mucosa

CRC vs NOR colorectal normal mucosa, patient 132	GSM1214423	tissue: normal mucosa	localisation: cecum	colorectal normal mucosa biopsy	female	cecum	normal mucosa
CRC vs NOR colorectal normal mucosa, patient 86	GSM1214424	tissue: normal mucosa	localisation: right colon	colorectal normal mucosa biopsy	female	right colon	normal mucosa
CRC vs NOR colorectal normal mucosa, patient 89	GSM1214425	tissue: normal mucosa	localisation: rectum / anal canal	colorectal normal mucosa biopsy	female	rectum / anal canal	normal mucosa

Table S6. The detailed clinical information for dataset GSE156355.

title	geo_accession	source_name_ch1	organism_ch1	characteristics_ch1	taxid_ch1	tissue type: ch1
CRC_Normal_Patient1	GSM4729410	Normal colon tissue, CRC Patient 1	Homo sapiens	tissue type: Normal	9606	Normal
CRC_Normal_Patient2	GSM4729411	Normal colon tissue, CRC Patient 2	Homo sapiens	tissue type: Normal	9606	Normal
CRC_Normal_Patient3	GSM4729412	Normal colon tissue, CRC Patient 3	Homo sapiens	tissue type: Normal	9606	Normal
CRC_Normal_Patient4	GSM4729413	Normal colon tissue, CRC Patient 4	Homo sapiens	tissue type: Normal	9606	Normal
CRC_Normal_Patient5	GSM4729414	Normal colon tissue, CRC Patient 5	Homo sapiens	tissue type: Normal	9606	Normal
CRC_Normal_Patient6	GSM4729415	Normal colon tissue, CRC Patient 6	Homo sapiens	tissue type: Normal	9606	Normal
CRC_Tumor_P	GSM4729	Tumor tissue,	Homo	tissue type:	9606	Tum

atient1	416	CRC Patient 1	sapiens	Tumor		or
CRC_Tumor_P atient2	GSM4729 417	Tumor tissue, CRC Patient 2	Homo sapiens	tissue type: Tumor	9606	Tum or
CRC_Tumor_P atient3	GSM4729 418	Tumor tissue, CRC Patient 3	Homo sapiens	tissue type: Tumor	9606	Tum or
CRC_Tumor_P atient4	GSM4729 419	Tumor tissue, CRC Patient 4	Homo sapiens	tissue type: Tumor	9606	Tum or
CRC_Tumor_P atient5	GSM4729 420	Tumor tissue, CRC Patient 5	Homo sapiens	tissue type: Tumor	9606	Tum or
CRC_Tumor_P atient6	GSM4729 421	Tumor tissue, CRC Patient 6	Homo sapiens	tissue type: Tumor	9606	Tum or
